# Oral inflammation promotes oral squamous cell carcinoma invasion

**DOI:** 10.18632/oncotarget.25540

**Published:** 2018-06-26

**Authors:** Cameron Goertzen, Hayder Mahdi, Catherine Laliberte, Tomer Meirson, Denise Eymael, Hava Gil-Henn, Marco Magalhaes

**Affiliations:** ^1^ Cancer Invasion and Metastasis Laboratory, Faculty of Dentistry, University of Toronto, Toronto, Canada; ^2^ Cell Migration and Invasion Laboratory, Faculty of Medicine in the Galilee, Bar-Ilan University, Safed, Israel; ^3^ Oral Pathology and Oral Medicine, Faculty of Dentistry, University of Toronto, Toronto, Canada; ^4^ Sunnybrook Health Sciences Centre, Toronto, Canada

**Keywords:** cancer, metastasis, inflammation, neutrophils, TNF

## Abstract

Oral squamous cell carcinoma (OSCC) represents 95% of oral malignancies and invasion, and metastasis underlies disease morbidity and mortality. We recently established a direct link between oral inflammation and cancer invasion by showing that neutrophils increase OSCC invasion through a tumor necrosis factor (TNFα)-dependent mechanism. The objective of this study was to characterize OSCC-associated inflammation and to determine the molecular mechanisms underlying inflammation-mediated OSCC invasion. Our results showed a significant increase in neutrophil infiltration, the neutrophil-to-lymphocyte ratio in the OSCC microenvironment and increased inflammatory markers, particularly TNFα in saliva. We performed next-generation sequencing of the TNFα-treated OSCC cells and showed marked overexpression of over 180 genes distributed among clusters related to neutrophil recruitment, invasion, and invadopodia. At the molecular level, TNFα treatment increased phosphoinositide 3-kinase (PI3K)-mediated invadopodia formation and matrix metalloproteinase (MMP)-dependent invasion. We show here that TNFα promotes a pro-inflammatory and pro-invasion phenotype leading to the recruitment and activation of inflammatory cells in a paracrine mechanism. Increased TNFα in the tumor microenvironment tips the balance towards invasion leading to decreased overall survival and disease-free survival. This represents a significant advancement of oral cancer research and will support new treatment approaches to control OSCC invasion and metastasis.

## INTRODUCTION

Oral squamous cell carcinoma (OSCC) represents the majority of oral cancers, and the poor outcome of this disease is attributed to late detection and presence of metastasis at the time of diagnosis [1]. For localized disease without metastasis, the 5-year survival rate is 80% but drops to 59% and 36% in regional and disseminated disease respectively [2]. This survival rate has not significantly improved in the last three decades emphasizing the need to better understand OSCC pathogenesis to increase patient survival and decrease morbidity [3].

Inflammatory cells are essential constituents of the microenvironment of cancers and can promote cancer cell proliferation and survival, as well as their ability to invade and metastasize [4, 5]. Several studies have shown that pro-inflammatory mediators, such as interleukin (IL)6, IL8, and tumor necrosis factor (TNFα), are elevated in oral cancer patients [6–8] and in the saliva of patients with pre-neoplastic oral lesions [9] but the mechanisms by which inflammation modulates oral cancer behavior are mostly unknown. Recently, we have established a new direct link between inflammation and oral cancer invasion by showing that neutrophils increase OSCC invasion, matrix degradation, and invadopodia formation, independent of direct contact, through a TNFα-dependent mechanism [10]. Invadopodia are actin-rich cellular protrusions formed by tumor cells which cause extracellular matrix degradation. The ability of tumor cells to undergo metastasis is closely associated with invadopodia formation [11]. The extracellular matrix degradation by invadopodia is mediated through matrix metalloproteinase (MMP) delivery and secretion [10, 12]. Clinically, increased expression of MMPs have been strongly associated with cancer progression, metastasis, and poor prognosis [11]. Therefore, it is crucial to investigate and understand the underlying processes of invadopodia formation in OSCC, specifically the role of oral inflammation, in order to prevent invasion and metastasis.

Here we performed a comprehensive examination of OSCC-associated inflammation *in vivo* and determined the molecular mechanisms underlying TNFα-mediated OSCC invasion *in vitro*. We described a novel analysis of patient samples using fluorescent immunohistochemistry (FIHC) and a novel semi-automated colocalization quantification to show an increase in the inflammatory cell infiltrate density, particularly neutrophils at the tumor microenvironment compared to non-neoplastic and pre-malignant lesions. Examination of saliva from OSCC patients demonstrated a significant increase in inflammation mediators, particularly TNFα. Next-generation sequencing and bioinformatics analysis corroborate the clinical findings showing that TNFα promotes up-regulation of genes that are associated with neutrophil recruitment, invadopodia, and invasion. Overall, TNFα stimulation promoted a pro-invasive and pro-inflammatory phenotype. Survival analysis showed that genes upregulated by TNFα stimulation are associated with reduced overall survival (OS) and disease-free survival (DFS) of OSCC patients.

We further analyzed the role of TNFα in cancer invasion using two-dimensional (2D) and three-dimensional (3D) invasion assays. We demonstrate that TNF-α promotes invadopodia formation, maturation and OSCC invasion in a phosphoinositide 3-kinase (PI3K) and Src-dependent mechanism. Our results support a novel mechanism by which neutrophils are recruited to OSCC, promoting TNFα secretion and leading to OSCC gene expressional changes and overall poor survival.

## RESULTS

### Increased recruitment of inflammatory cells in OSCC

**Figure 1:**
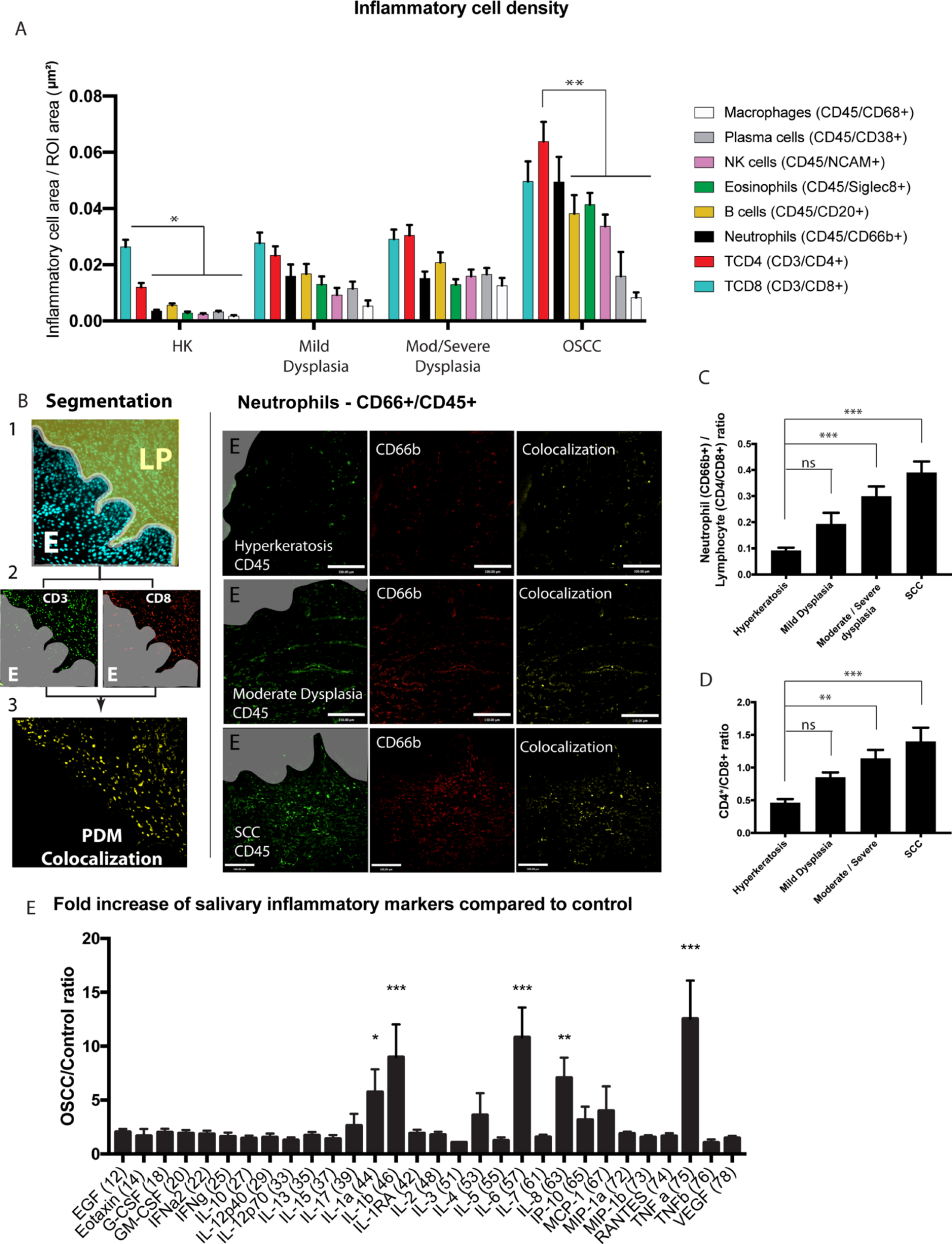
Increased recruitment of inflammatory cells in primary OSCC tumors (**A**) Inflammatory cell area (µm^2^) standardized to a region of interest (ROI) in samples from patients diagnosed with hyperkeratosis (HK), mild dysplasia, moderate/severe dysplasia, or OSCC. The inflammatory cells were identified by semi-automated colocalization of 2 markers for each cell using FIHC. *n* = 39 samples total (hyperkeratosis *n* = 9, mild dysplasia *n* = 9, moderate/severe dysplasia *n* = 10, OSCC *n* = 10). (**B**) Left panel: Representative image showing the steps of data analysis – 1- segmentation in epithelium-E and lamina propria-LP, 2- colocalization and 3- quantification. Right panel: Representative images of patient samples diagnosed with hyperkeratosis, moderate dysplasia, and OSCC showing colocalization (yellow, overlay) of CD45 (green) and CD66b (red). Scale bar, 100 μm. Gray areas represent the epithelium or OSCC identified in the DAPI channel. **(C)** Ratio of neutrophil (CD66b+) to lymphocyte (CD4+ and CD8+). The ratio was calculated using the normalized inflammatory cell area of neutrophils divided by the combined CD4 and CD8 positive inflammatory area in each sample as described in panel A. Similarly, CD4 inflammatory cell area divided by CD8 in each sample was used to calculate the CD4/CD8 ratio (**D**). **(E)** The salivary inflammatory markers were quantified using a Multiplexing Luminex based assay. Saliva was collected from 13 control patients and 17 OSCC patients as described in the materials and methods section. The results are normalized to control samples. Inflammatory area or ratios are presented as columns ± SEM. One-way ANOVA followed by Dunnett’s multiple comparison test: ^*^P < 0.05; ^**^, *P* < 0.01; ^***^, *P* < 0.001.

OSCC is commonly preceded by a range of tissue and cellular alterations. These conditions are termed oral epithelial dysplasia, and are subdivided based on specific histological criteria as mild, moderate and severe and further classified [17, 18]. Since the recommended treatment of dysplasia is binary (mild dysplasia is monitored while moderate/severe are surgically removed), we clustered moderate and severe dysplasia in our FIHC analysis. To determine whether neutrophils and other inflammatory cells are recruited to potentially malignant (or pre-malignant) lesions and OSCC *in vivo*, we analyzed non-neoplastic (NN) (hyperkeratosis, no dysplasia), potentially malignant (PM) (mild dysplasia and moderate/severe dysplasia) and OSCC samples from 39 patients. We used a novel semi-automated colocalization analysis technique to determine the inflammatory cell population associated with NN, PM and OSCC lesions (Figure 1A). Using this new technique, we could consistently quantify TCD4, TCD8, neutrophils, eosinophils, B cells, NK cells, macrophages and plasma cells in tissue samples (Figure 1B left panels). Our results show an increase in the overall inflammatory infiltrate from hyperkeratosis to dysplasia to OSCC samples (Figure 1A) as revealed through FIHC IHC imaging (Figure 1B, Supplementary Figure 1A, 1B). OSCC showed the highest density of inflammation for all cells except plasma cells and macrophages which were similar compared to dysplasia (mild, moderate/severe). Further analyses revealed a progressive, significant increase in neutrophil/lymphocyte ratio (Figure 1C) and TCD4/TCD8 ratio (Figure 1D) in OSCC compared to hyperkeratosis (non-neoplastic). Overall the results show a progressive increase in inflammation density, as well as changes to the quality of the, infiltrate from benign samples to OSCC. It also suggests that dysplasia-associated inflammation is a transitional stage between non-neoplastic mucosa and OSCC.

### Increased cytokines in the saliva of OSCC patients

To determine if cytokine expression is altered in the microenvironment of OSCC, we analyzed the cytokine expression in the saliva of 17 OSCC cancer patients and compared to 13 control patients without cancer or significant oral diseases (See Supplementary Table 3 for demographics). Human Cytokine Array analysis showed that cancer patients have a significant increase in saliva expression of pro-inflammatory markers IL-1a, IL-1b, IL-6, IL-8, and TNFα (Figure 1E) compared to controls. Other cytokine markers were slightly elevated in the saliva of cancer patients compared to control patients but were not statistically significant (Supplementary Figure 1C–1E). This is consistent with our results showing a consistent increase in the inflammatory infiltrate, particularly neutrophils and TCD4 cells in OSCC patients (Figure 1A)

### TNFα stimulation of OSCC cells promotes up-regulation of gene clusters associated with neutrophil recruitment, invadopodia, and invasion.

**Figure 2:**
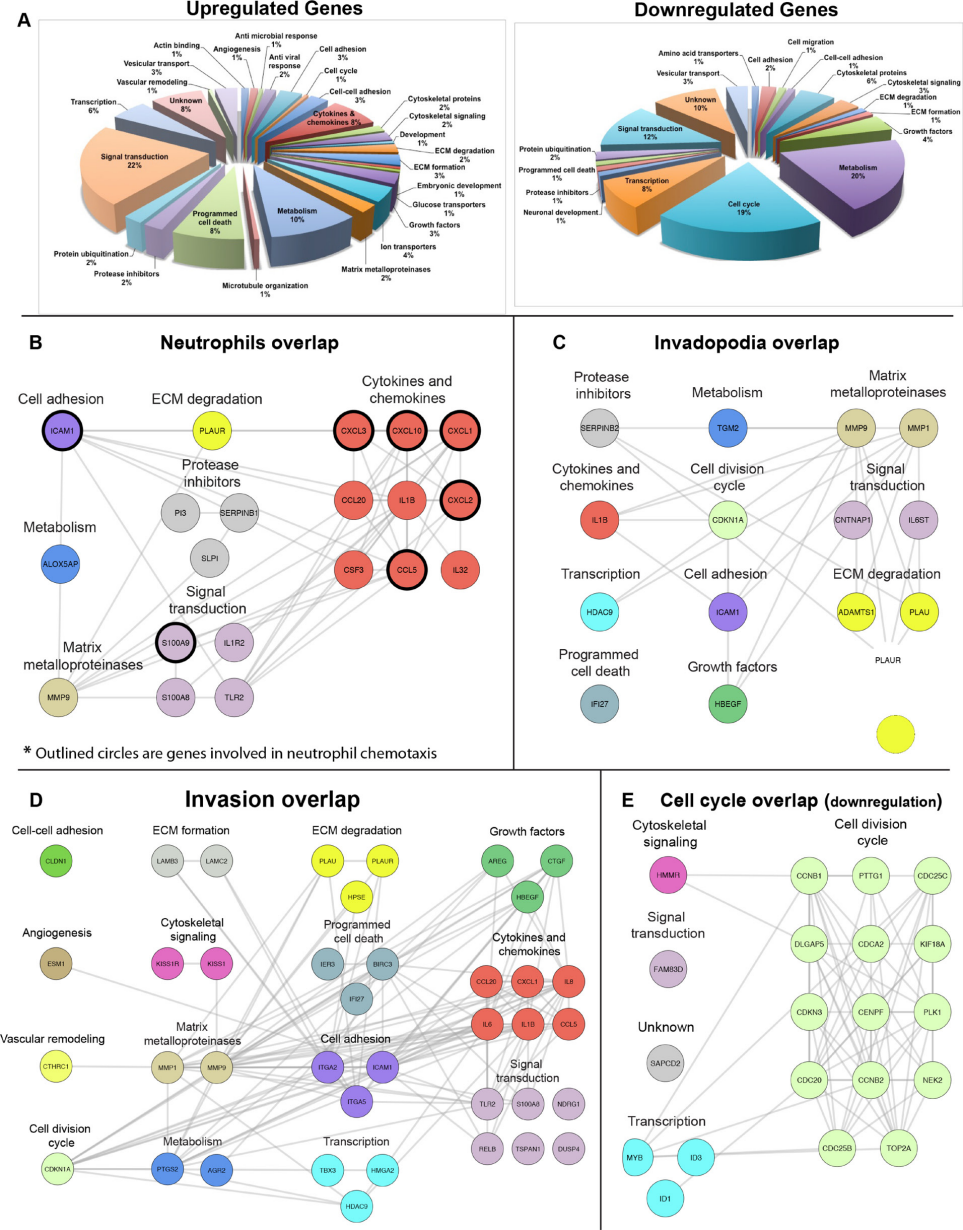
TNFα stimulation of OSCC cells promotes up-regulation of gene clusters associated with neutrophil recruitment, invasion, and invadopodia **(A)** Classification of up-regulated and down-regulated genes identified by mRNA sequencing, based on their GeneCards and UniProt identified function. Overlap of known neutrophil-related genes (genes which products can recruit/activate neutrophils) (**B**), invadopodia (**C**), and invasion **(D**) up-regulated genes and cell-cycle (**E**) down-regulated genes identified by mRNA sequencing. The lists of genes were overlaid using Cytoscape, and the combined list of common nodes was used to build a physical and functional map association in STRING (grey lines). The results are based on three independent experiments in which 500,000 UMSCC1 cells were stimulated with TNFα (10 ng/mL) followed by lysis and RNA extraction.

Our previous results demonstrated that neutrophils promote cancer invasion through a TNFα- dependent pathway [19] and our data (Figure 1) now shows a significant increase in neutrophils and TNFα in the saliva of cancer patients (Figure 1). To gain insight into the molecular mechanism by which invasion is induced in OSCC, we performed mRNA sequencing of UMSCC1 cell line stimulated by TNFα. Our analysis revealed a significant, at a minimum, two-fold increase in the expression of 180 different genes (Supplementary Table 4) and significant reduction of over 80 genes (Supplementary Table 5). Gene ontology analysis of up-regulated genes using DAVID revealed enrichment in several signaling pathways including TNF signaling pathway, NFκB pathway, cytokine-mediated signaling, and inflammatory response and a significant change in cell cycle associated genes in the down-regulated mRNA group (Figure 2A, Supplementary Figure 2A). A hypergeometric test revealed a significant increase in neutrophil, invasion, and invadopodia associated genes in the up-regulated mRNA group (Supplementary Figure 2B). To identify specific genes within the up-regulated group that are involved in neutrophil function, invasion, and invadopodia we performed literature mining using GLAD4U and ALS databases with the relevant queries. Overlays of the combined literature mining genes with up-regulated mRNA sequencing data revealed 20 neutrophil-related genes that are expressed by cancer cells and the products are involved in neutrophil chemotaxis and activation (Figure 2B, Supplementary Figure 3A), 15 invadopodia-related genes (Figure 2C, Supplementary Figure 3B), and 39 invasion-related genes (Figure 2D, Supplementary Figure 3C). Overlays of the combined literature mining genes with down-regulated mRNA sequencing data revealed 20 cell cycle-related genes (Figure 2E, Supplementary Figure 3D). Likewise, gene mining using IPA, UniProt, and GeneCard revealed gene expression location to occur ubiquitously throughout the cell (Supplementary Figure 2C). These results are in keeping with our clinical analysis showing increased neutrophils and IL-1a, IL-1b, IL-6, IL-8, and TNFα in OSCC patients (Figure 1).

Among the genes that showed most overexpression (over 10-fold change), IL8 (12.5 fold increase) and MMP9 (12.5 fold increase) have been shown recently to be involved in the neutrophil-induced increase of OSCC invasion in a TNFα-dependent pathway [10]. We have confirmed the sequencing results by measuring changes in IL8 and MMP9 concentration in the supernatants of UMSCC cells (UCSCC1, UCSCC2, UCSCC43, and UCSCC47). Maximum changes were achieved through TNFα treatment of concentration 10 ng/ml, and hence all following experiments were carried out at this concentration (Supplementary Figure 4A). TNFα treatment of UMSCC1 cells caused a significant increase in IL8 (Supplementary Figure 4B) and MMP9 (Supplementary Figure 4C) expression in the cell supernatant.

### TNFα stimulation promotes oral cancer invasion

**Figure 3:**
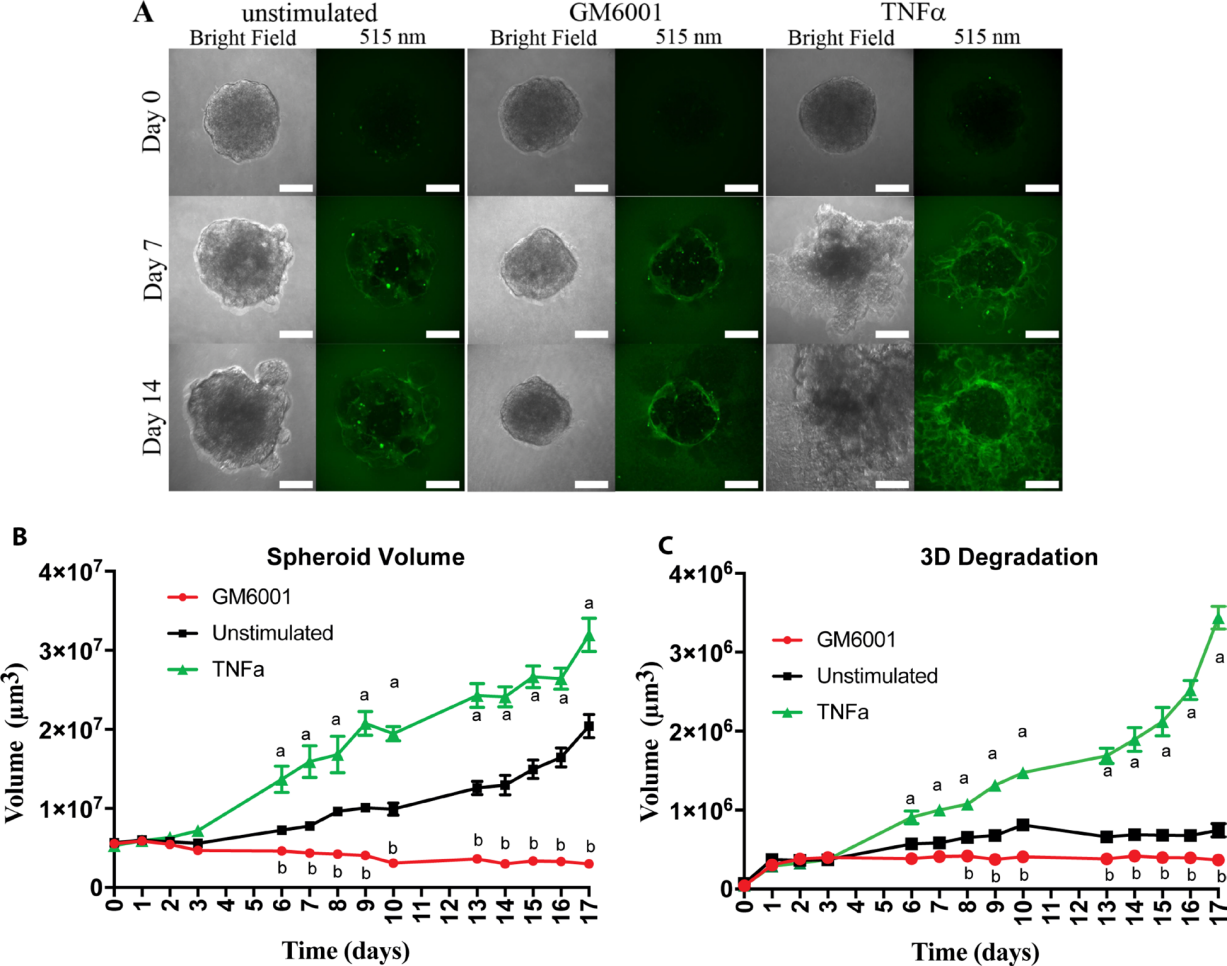
TNFα stimulation of OSCC cells leads to increased matrix degradation and spheroid volume (**A**) Representative confocal images of UMSCC1 formed spheroids at Day 0, 7, and 14. Spheroid volume was revealed through bright-field imaging and Geltrex degradation through 505 nm excitation of BSA-Green degradation marker. Scale bar, 100 μm. **(B)** Spheroid volume over a period of 17 days in the presence or absence of TNFα (10 ng/mL) or GM6001 (25 µM). (**C**) Geltrex degradation volume over a period of 17 days in the presence or absence of TNFα (10 ng/mL) or GM6001 (25 µM). Volumes are presented as mean per spheroid ± SEM. Two-way ANOVA followed by Bonferroni post hoc test: a, *P* < 0.01 TNFα vs. unstimulated; b, *P* < 0.01 for GM6001 vs. unstimulated; *n* = 3.

Our previous study showed that neutrophils increase OSCC invasion in a TNFα-dependent mechanism [10]. Our genetic analysis (Figure 2) showed that TNFα leads to up-regulation of genes associated with invadopodia activation and invasion. To further determine the mechanisms by which TNFα leads to poor survival, we measured OSCC invasion using a novel three-dimensional (3D) assay. Our spheroid invasion assay combines invasion and degradation in 3D to better mimic the tumor microenvironment seen *in vivo*. Briefly, tumor spheroids were formed and grown in Geltrex, a synthetic basement membrane matrix, containing DQ-Green BSA, which upon degradation by proteases results in green fluorescent signaling. Spheroid volume and Geltrex degradation were observed to increase over the 17-day observation period in the unstimulated control, and TNFα stimulated conditions but appeared to remain constant with the addition of the pan MMP inhibitor, GM6001 (Figure 3A). Stimulation of the spheroids with TNFα resulted in a significant increase in both spheroid volume and degradation volume (Figure 3B and 3C) compared to unstimulated control, while the addition of GM6001 significantly decreased degradation and spheroid volume (Figure 3B and 3C).

### TNFα stimulation of OSCC cells promotes invadopodia formation

**Figure 4:**
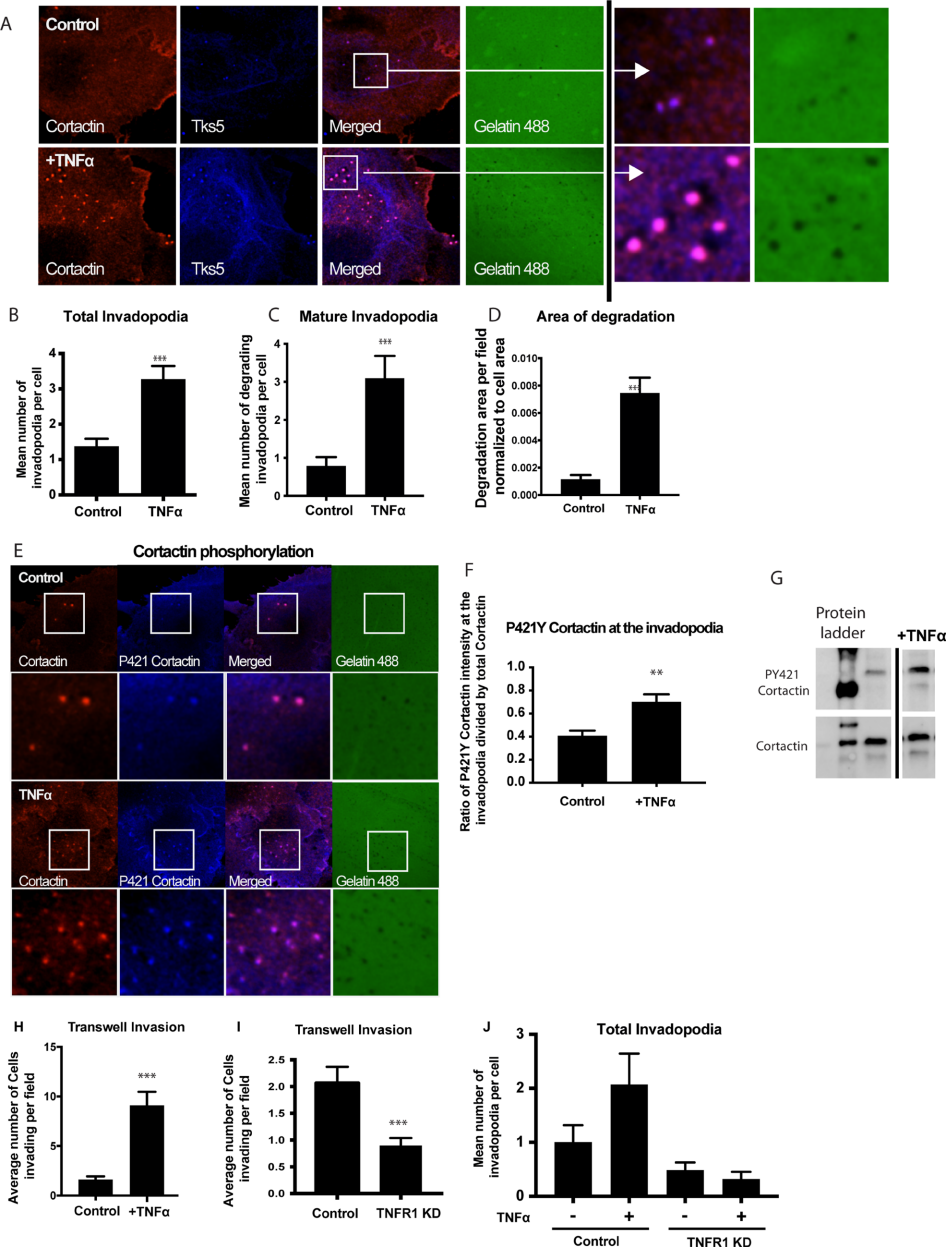
Treatment with TNFα stimulates invadopodium formation in UMSCC1 cells UMSCC1 cells were plated on a thin gelatin matrix and incubated for 24 hours in the presence or absence of TNFα (10 ng/mL). (**A**) Representative images of UMSCC1 cells forming invadopodia in green fluorescence gelatin - cortactin (red), Tks5 (blue) and gelatin (green). The total number of invadopodia (**B**) and mature invadopodia (**C**) per cell were counted. Area of gelatin degradation was measured in the while field and normalized to the area of the cell in the field (**D**). Representative images of cortactin phosphorylation in invadopodia formed in UMSCC1 cell in the presence or absence of TNFα (10 ng/mL). Cortactin (red), P421 Cortactin (blue), gelatin (green) (**E**). We have calculated the ratio of mean pixel intensity (MFI) of phosphorylated P421Y cortactin at the invadopodia in the presence or absence of TNFα (10 ng/mL) and divided by the MFI of total cortactin in the same pixel, and the results are shown in (**F**). A total of 76 cells were analyzed in 3 experiments. Representative western blot of cortactin tyrosine residue 421 phosphorylation and total cortactin expression after TNFα stimulation. (**G**). For transwell experiments, we calculated the average number of UMSCC1 cells invading per Transwell imaging field (**H**) in UMSCC1 cells with TNFR1 knockdown (**I**) and the formation of invadopodia (**J**) in the presence or absence of TNFα (10 ng/mL). One-way ANOVA followed by Dunnett’s multiple comparison test: ^**^P < 0.01; ^***^, *P* < 0.001. *n* = 3 for invadopodia experiments and western blot, *n* = 5 for transwell experiments.

To determine the role of TNFα in an invadopodia-dependent invasion, we evaluated invadopodia formation and maturation in TNFα-treated UMSCC1 cells as indicated by colocalization of invadopodia markers, cortactin, and Tks5, over degraded gelatin (Figure 4A). Upon stimulation with TNFα, there was a significant increase in the total number of invadopodia formed per cell (Figure 4B) as well as the number of mature invadopodia (Figure 4C) and the total area of invadopodia-mediated matrix degradation (Figure 4D). There were no significant changes to the average size of the invadopodia (Control 1.75 ± 0.08 µm^2^ and TNF stimulated was 1.90 ± 0.13 µm^2^). Cortactin phosphorylation is a required step in invadopodia maturation; therefore we analyzed levels phosphorylation at cortactin tyrosine residue 421 (p421Y) after TNFα stimulation. Our results showed a significant increase in cortactin phosphorylation after TNFα stimulation at the invadopodia and cell lysates (Figure 4E–4G). To further understand the role of TNFα in OSCC, invadopodia assays and Transwell-based invasion assays were performed using UMSCC1 cells with TNFα receptor (TNFR1) knockdown by siRNA. The results demonstrated that TNFR1 knockdown resulted in a decrease in UMSCC1 invasion and complete inhibition of TNFα-induced invadopodia formation (Figure 4H–4J). Altogether, we showed that TNFα stimulation through its receptor, TNFR1, promotes a significant increase in OSCC invasion.

### TNFα induced invasion requires PI3K

**Figure 5:**
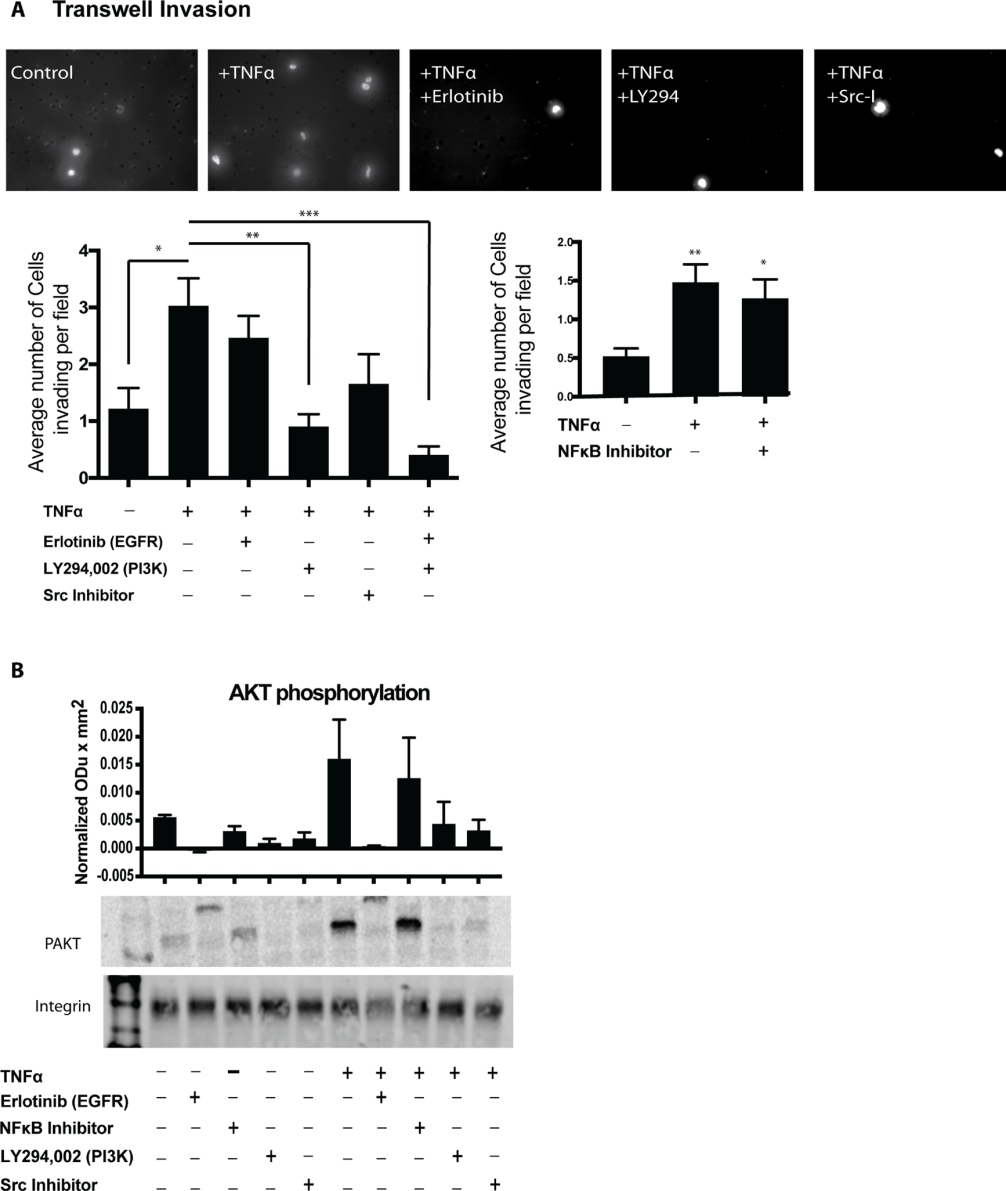
PI3K and Src inhibition lead to decreased OSCC invasion (**A)** UMSCC1 cells were plated in the upper chamber and incubated in the presence or absence of TNFα (10 ng/mL) without or with inhibitors. Cell invasion was calculated as the average cell coverage area under the membrane after 24-hour incubation. Two-way ANOVA followed by Bonferroni post hoc test: ^*^P < 0.05 for TNFα (10 ng/mL) control vs. unstimulated control. ^**^, *P* < 0.01; ^***^, *P* < 0.001 for inhibitor treatment vs. TNFα (10 ng/mL) control. (**B**) Representative western blot showing the expression of PAKT in UMSCC1 cells following TNFα (10 ng/mL) stimulation without or with inhibitors. Representative blot of 3 experiments. Columns represent the intensity of phospho-AKT ± SEM. One-way ANOVA followed by Dunnett’s multiple comparison test: ^*^P < 0.05; ^**^, *P* < 0.01; ^***^, *P* < 0.001

As revealed through the gene classification analysis, TNFα stimulation induces the overexpression of multiple genes associated with PI3K-AKT signaling as well as EGFR activation. To determine the mechanism by which TNFα stimulates OSCC invadopodia formation and invasion, we utilized Transwell invasion assays in OSCC cells treated with inhibitors for EGFR (erlotinib), NFκB (sc-3060), Src (sc-204303) and PI3K (Lyg294). There was a marked decrease in invasion following the inhibition of PI3K (Figure 5A) while the addition of both Erlotinib and PI3K-inhibitor suggested an additive effect. Src and EGFR inhibition individually showed a trend towards decreased invasion (Figure 5A). We further investigated these findings by analyzing the phosphorylation levels of AKT following stimulation with TNFα. As seen in Figure 5B, TNFα induced a 3-fold increase in phosphorylated S473 AKT compared to the non-stimulated control. Inhibition of EGFR, PI3K, and Src blocked TNFα-induced AKT phosphorylation. Our results show that TNFα-induced invasion requires PI3K activation in a mechanism independent of EGFR activation or NFκB transcription regulation. We also analyzed IL8 expression after the addition of the above inhibitors and showed a significant reduction in IL8 expression both in unstimulated, and TNFα treated cells after Src, PI3K and EGFR inhibition (Supplementary Figure 4D).

### Increased expression of TNFα-stimulated genes correlates with poor patient survival

**Figure 6:**
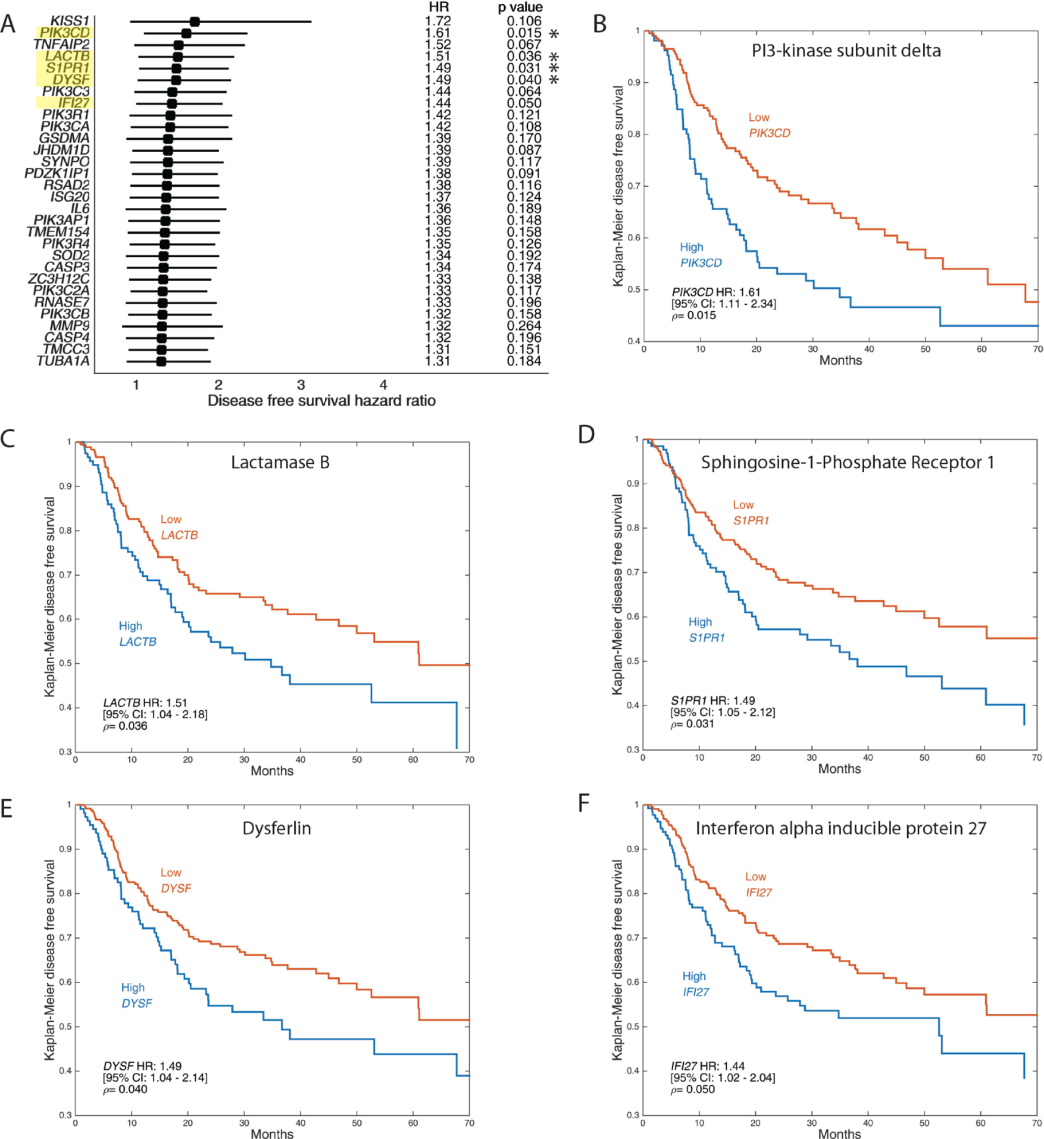
Up-regulation of invasion and invadopodia-related genes is correlated with poor OSCC patient disease-free survival RNA sequencing data for 519 head and neck squamous cell carcinoma (HNSCC) samples were retrieved from The Cancer Genome Atlas (TCGA) and analyzed with cBioPortal and MATLAB. The analyzed dataset contains mRNA expression Z-scores (RNA-Seq V2 RSEM) computed as the relative expression of an individual gene and tumor to the expression distribution of all samples that are diploid for the gene. Samples were split into high and low expressing groups based upon mRNA expression Z-scores. The Z-score cut-off value for a given gene was defined as that which gave the lowest *p*-value. (**A**) Forest plots of top 30 DFS genes obtained by comparing the up-regulated genes that appeared by mRNA sequencing data to TCGA database as above. Kaplan–Meier representation of disease-free survival in 519 HNSCC samples from TCGA database correlated to expression of genes PI3-Kinase subunit delta (**B**), lactamase B (**C**), sphingosine-1 phosphate receptor 1 (**D**), Dysferlin (**E**), and interferon alpha-inducible protein 27 (**F**).

To evaluate the relevance of up-regulated genes in our mRNA sequencing analysis to head and neck squamous cell carcinoma patient prognosis, we overlapped the up-regulated genes obtained in our analysis to RNA sequencing data from 519 HNSCC samples obtained from TCGA and analyzed disease-free survival (Figure 6A) and overall survival (Supplementary Figure 5) of patients in correlation with low expression versus high expression of the relevant genes. Our analysis showed that TNFa induced the expression of several genes that correlated with decreased disease-free survival (DFS) and overall survival (OS) across all cases of HPV-negative OSCC (including smokers). Genes that significantly decrease DFS include PI3-Kinase subunit delta (Figure 6B), lactamase B (Figure 6C), sphingosine-1 phosphate receptor 1 (Figure 6D), Dysferlin (Figure 6E), and interferon alpha-inducible protein 27 (Figure 6F). Genes that significantly decrease OS include Gasdermin A (Supplementary Figure 5B), superoxide dismutase 2 (Supplementary Figure 5C), gap junction beta-2 protein (Supplementary Figure 5D) and interferon alpha-inducible protein 27 (Supplementary Figure 5E).

## DISCUSSION

Inflammatory cells and mediators are present in the microenvironment of most of the tumors but the mechanisms of inflammation in cancer progression, particularly in invasion and metastasis, were only recently recognized [5, 19]. Here we showed a progressive, significant increase in inflammatory infiltrate density as well as qualitative changes in the specific cell populations in pre-malignant and OSCC samples causing a significant increase in TCD4/TCD8 ratio and neutrophil/lymphocyte ratio compared to non-neoplastic hyperkeratosis. Both these changes have been reported in peripheral blood samples, but this is the first report of such changes in tumor specimens [20]. Likewise, examination of OSCC patients’ saliva samples revealed a significant increase in pro-inflammatory cytokines IL-1a, IL-1b, IL-6, IL-8, and TNFα. In our results, TNFα was the highest fold increase compared to control both of which is consistent with the increase in Neutrophils seen in the tumor stroma and supports our previous observation that OSCC and neutrophils develop a paracrine co-stimulatory loop [10]. Jou *et al.* previously reported similar salivary biomarkers to correlate with OSCC tumor stage and demonstrated the capacity of these markers to be used in the diagnosis of human oral cancers [21]. Hence, these markers identified by FIHC and saliva analysis may be potentially utilized as markers to predict the outcomes of OSCC patients.

Neutrophils co-cultured with OSCC cells increased OSCC invasion through a TNFα dependent process but the mechanism by which TNFα stimulates OSCC invasion remains unclear [10]. Our sequencing results revealed that TNFα plays a crucial role in the development of a pro-invasion tumor phenotype through the up-regulation of genes involved in several cancer pathways, particularly matrix remodeling and inflammation. Interestingly, an overlay of combined literature mining using cell cycle query with down-regulated mRNA expression data showed an overlap of 20 genes, among them 14 cell division cycle associated genes and three genes involved in transcription. Additional illustration using Ingenuity Pathway analysis revealed a significant fraction of genes encoding for extracellular cytokines, chemokines, and metalloproteinases that are highly expressed by OSCC cells following TNFα stimulation. Several pro-inflammatory genes were up-regulated, including cytokines containing the C-X-C motif such as CXCL1, CXCL3, CXCL2, and CXCL10, which have been previously reported to promote OSCC progression [22, 23]. Likewise, our analysis revealed an upregulation of genes S100A8 and S100A9 which have previously been reported as salivary biomarkers of OSCC, whose expression has been shown to induce tumor-infiltrating monocytes and macrophages leading to increased cancer cell invasion and metastasis [21, 24]. In summary, our sequencing findings establish TNFα as a significant promoter of cancer progression, modulating the gene expression in such a way that oral cancer cells decrease cell division and increase invasion.

Utilization of 3D *in vitro* invasion assays has shown to be feasible and to better represent the conditions that dictate cancer invasion as seen *in vivo* [12, 25, 26]. Through the novel approach of adding the degradation marker, DQ-BSA, green to the reconstituted basement membrane matrix, we could measure and compare tumor spheroid invasion/and degradation. Our findings demonstrate that the addition of TNFα dramatically increased tumor invasion and growth. The elevation of tumor invasion is supported by our sequencing results that demonstrated TNFα up-regulates invasion-related genes of MMP1, MMP9, MMP14, LAMB3, and FGF2 which all have been previously reported to increase OSCC cancer invasion [27–29]. Likewise, we also showed that TNFα up-regulate genes CLDN-1, IL6, and NFκBIA, which have previously been reported to increase OSCC cell proliferation, supporting our increased tumor volume findings [30–32].

Our recent results show that neutrophils increase OSCC invadopodia formation and that OSCC co-cultures supernatants show a significant increase in IL8 and TNFα expression compared to isolated cultures [10]. Our findings here show that TNFα stimulation increases IL8 and MMP9 secretion by OSCC cells, supporting our previous findings and reinforcing our sequencing results demonstrated here. More so, we demonstrate that the TNFα receptor, TNFR1, plays a important role in the maturation and function of invadopodia. Previous studies have demonstrated the importance of receptor localization/activation in the formation and activation of invadopodia as reported with receptor families G-protein-coupled receptors (GPCRs), epidermal growth factor receptors (EGFRs), and receptor tyrosine kinases (RTKs) [12, 33, 34]. To the best of our knowledge, this is the first demonstration of TNFR1 knockdown in OSCC cells leading to decrease in invadopodia formation suggesting a direct role for the TNFα in the activation of invadopodia and basement membrane degradation through MMP9 release.

To determine the mechanism by which TNFα stimulates OSCC invasion, we utilized inhibitors of molecules involved invadopodia formation. Firstly, our sequencing results demonstrated that TNFα treatment increased genes that regulate EGFR activation such as HB-EGF or AREG [35–37]. Hence, to determine the role of EGFR downstream of TNFα in OSCC cells, we utilized the inhibitor erlotinib, a receptor tyrosine kinase inhibitor which acts to inhibit EGFR function. Our findings demonstrate that TNFα requires EGFR to increase OSCC IL8 expression and secretion. This is supported by a previous study in which Hwang *et al.* demonstrated in OSCC cells, that IL8 expression and secretion was dependent on the EGFR signaling pathway [38]. More so, our findings demonstrate that TNFα induced AKT phosphorylation which is dependent on PI3K and Src, both of which have been previously reported as necessary for cytokine expression [39, 40]. Furthermore, our findings demonstrate that TNFα requires NFκB, Src kinase and PI3 kinase to induce OSCC invasion. Rehman and Wang showed that in OSCC, C-X-C chemokine receptors activate NF-κB leading to increased OSCC cell invasion [41]. Lastly, it is well established that Src is a key player in invadopodia formation [12, 14, 42–44]. Likewise, PI3K is reported to induce invadopodia formation and cell invasion in several cancers including lung and breast [45, 46]. Therefore, we put forward that TNFα signals through Src and PI3K-dependent pathways leading to increased invadopodia formation and OSCC cell invasion as our findings suggest.

**Figure 7:**
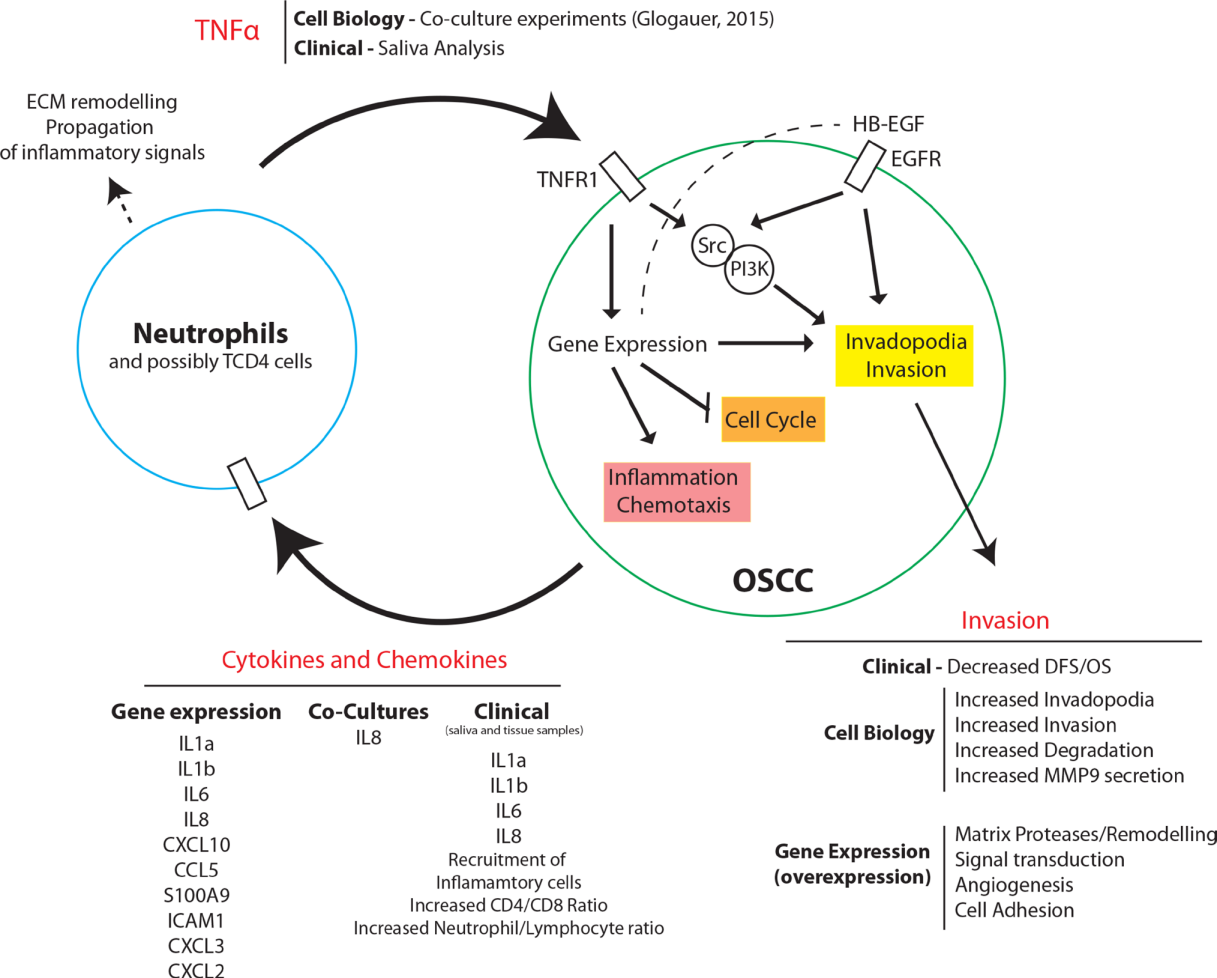
Proposed model for TNFα-mediated OSCC invasion Schematic diagram of TNFα signaling in OSCC invasion. TNFα released by neutrophils during oral inflammation signals via TNFR1 to induce gene expressional changes, invadopodia formation, and inflammatory cytokine and chemokine release. TNFR1 mediated PI3K and Src activation, heightened by EGFR signaling, leads to invadopodia formation, MMP9 secretion, ECM degradation, and invasion. Additionally, TNFR1 signaling leads to down-regulation of cell cycle-related genes. TNFR1 induced up-regulation of genes associated with inflammation and chemotaxis leads to cytokine and chemokine release by OSCC cells and acts through paracrine signaling and activation of nearby neutrophils. Active neutrophils promote increased inflammatory signaling and TNFα release, completing a positive feedback loop with OSCC cells.

Overall, our results demonstrate a novel mechanism by which TNFα stimulates OSCC invasion (Figure 7). Based on our results, we propose that oral chronic/acute inflammation leads to a “pro-tumor” phenotype by recruiting neutrophils that will establish paracrine activation with OSCC cancer cells. In combination with our previous findings, we suggest that TNFα is released by neutrophils during oral inflammation which then signals *via* TNFR1 on the OSCC cell membrane to induce gene expression changes and PI3K and Src kinase activity. PI3K and Src activation lead to downstream signaling to induce invadopodia formation and invasion. Likewise, TNFα signaling through TNFR1 and its downstream signaling factors leads to upregulation of genes related to invasion, inflammation and neutrophil recruitment, and downregulation of genes related to cell cycle. Upregulation of genes related to invasion, such as MMP9, increases basement membrane degradation and extracellular matrix remodeling by OSCC. Additionally, the pro-inflammatory phenotype of OSCC is conferred through TNFα mediated increase of cytokines and chemokines released by the OSCC cell, such as IL8, which acts through paracrine signaling, recruiting and binding to nearby neutrophils. This increases CD4/CD8 and neutrophil/lymphocyte ratios as clinically observed. Recruitment of neutrophils and activation by cytokines leads to increased TNFα expression which then activates TNFR1 on the OSCC cells, completing a positive-feedback loop.

Increased neutrophil infiltrate in OSCC has been previously suggested to correlate with poor patient survival. Shen *et al.* showed that the presence of intratumoral neutrophils is an independent prognostic factor for disease-free survival, cancer-specific survival and overall survival [47]. Hence, here we examined whether inflammatory infiltrate and subsequent TNFα release in the OSCC microenvironment effects patient survival. Our analysis of TNFα up-regulated genes, compared to data from 519 HNSCC samples obtained from TCGA, identified several genes that correlated with decreased disease-free survival (DFS) and decreased overall survival (OS) some of which have been previously associated with cancer progression. Our results identify a complex mechanism in which inflammation promotes OSCC progression and suggest that the characterization of the inflammatory response can be used to predict outcome in OSCC. Suggests that the identification of inflammatory infiltrate in OSCC can be used to predict patient survivability. Our findings in this study may lead to fundamental changes to OSCC prevention and treatment by highlighting the importance of local control of chronic inflammation as a strategy to prevent OSCC invasion. Likewise, our results establish immunomodulatory mechanisms which can be used to develop new individualized treatments to block OSCC invasion and metastasis and improve patient survivability.

## MATERIALS AND METHODS

### Fluorescent immunohistochemistry (FIHC) of cancer patients

All methods and experiments are following the University of Toronto research guidelines. The experiments involving patients were approved by the University of Toronto research ethics board (REB) (protocols 00032723 and 00032763) and Sunnybrook Health Sciences Centre REB 223-2015.

### Study population

We performed a retrospective analysis of a total of 39 patient samples divided into four groups based on diagnosis (hyperkeratosis, mild dysplasia, moderate dysplasia/severe dysplasia, oral squamous cell carcinoma). The slides were selected from the archives of the Oral Pathology Department at the Faculty of Dentistry, University of Toronto and consisted of 9 cases of mild dysplasia, 10 cases of moderate/severe dysplasia and 10 cases of OSCC. The control group included nine oral biopsies of non-neoplastic/non-inflammatory lesion with the diagnosis of hyperkeratosis (age and gender-matched patients with no history of oral cancer). Samples were restricted to the lateral border of the tongue. The investigator did not have access to the diagnosis and was blinded to the samples’ details during the imaging and data collection stage.

### Fluorescent immunohistochemistry

8 unstained slides were obtained per case. The slides were deparaffinized using Xylene and sequential ethanol incubations followed by antigen retrieval in Citrate buffer, 98° C for 20 minutes. Samples were blocked with 1% bovine serum albumin (BSA), 10% normal serum for 2 h, followed by incubation with primary antibodies at 4° C. The antibodies used in this study are found in Supplementary Table 1. The combination of markers used to determine the inflammatory cell populations are found in Supplementary Table 2.

### Data analysis

The slides were analyzed using a Quorum Spinning Disk Confocal microscope (Quorum Technologies Inc., Canada) and the cells were identified based on the colocalization of the markers listed in Supplementary Table 2. The colocalization was performed through Volocity 3D Image Analysis Software (PerkinElmer, U.S.A) using staining intensity correlation and automatically generated thresholds based on Costes *et al.* (2004) and Li *et al.* (2004). First, the number of pixels with red [R] and green [G] intensities was plotted by the software as a scatterplot with each axis representing the intensity of each color as the product of the difference of [R] and [G] pixels from its respective mean (PDM). Subsequently, the software applies an algorithm that determines automatically the red and green channels’ thresholds [TR] and [TG] along a line whose slope and intercept (α and b) are obtained by linear least-square fit of the red and green intensities [IR] and [IG] over all pixels in the image (IG = α × IR + b). For hyperkeratosis and epithelial dysplasia lesions, the Region of Interest [ROI] was manually selected to represent the lamina propria of the connective tissue. Carcinoma lesions had islands and cords of epithelial cells invading the adjacent connective tissue. Here, we decided to use the cancer stroma as the ROI. All the subsequent data analysis was restricted to the ROI. The walls and the lumen of the medium-sized blood vessels within the lamina propria were removed from the ROI (Figure 1B). Please see Supplementary Table 1 for all antibodies and inhibitors used in this study.

### Salivary inflammatory mediators

A total of 17 cancer patients and 13 control patients without cancer or significant oral diseases (including advanced periodontal disease or immune-mediated disorders) were selected for this study. Patients with proven OSCC were recruited from the Odette Cancer Centre at the Sunnybrook Health Sciences Centre and provided their saliva by rinsing with 3 ml of cold 0.9% Saline solution for 30 seconds. Samples were centrifuged at 2600 g for 5 minutes, and the supernatant was collected. Protease inhibitor cocktail was added at a concentration of 1 µL/mL, and samples were placed on –80° C until analysis. The samples were analyzed by Luminex multiplexing using Millipore Human Cytokine/Chemokine Magnetic Bead panel protocol. Magnetic beads (25 µL), assay buffer (25 µL) and sample (25 µL) (1:2 diluted sample) were incubated overnight at 4° C with shaking. Beads were subsequently washed 2× times and incubated with 25 µL of detection antibody for 1 hour. 25 µL streptavidin-phycoerythrin was added to the assay mixture for 30 minutes at room temperature. Beads were washed 2× and re-suspended in 150 µL of Sheath Fluid. Assays were read with Luminex 100 Reader and data were analyzed using Bio-plex Manager 6.0. This study was approved by the University of Toronto Research Ethics Board and Sunnybrook Hospital research ethics board.

### Bioinformatic analysis and literature mining

Gene classification of biological processes, UniProt keywords, KEGG, and BioCarta pathway enrichment analysis was carried out for up-regulated and for down-regulated genes using DAVID [13]. GeneCards and UniProt databases were used to classify proteins from mRNA sequencing data (two-fold increase over control and two-fold decrease over control) by manual curation.

Hypergeometric test between the number of overlapping nodes for each term (neutrophil, invasion, invadopodia, or cell cycle) within up-regulated or down-regulated genes was performed using MATLAB. Comparative literature mining was conducted using two different automated literature-mining tools, Gene List Automatically Derived for You (GLAD4U) and Agilent Literature Search (ALS). GLAD4U search was performed using one of the queries “invasion/invadopodia/neutrophil/cell cycle” and limited to human context with a threshold of 0.01. ALS search was performed through Cytoscape using one of the queries “invasion/invadopodia/neutrophil/cell cycle” and limited to *Homo sapiens* with limited interaction lexicon. The combined lists of proteins identified by GLAD4U and ALS (a total of 1066 non-redundant genes for invasion, 426 genes for invadopodia, 286 genes that affect neutrophils function (neutrophils), and a total of 1123 non-redundant genes for cell cycle) and the lists of proteins identified by mRNA sequencing (a total of 180 up-regulated genes or 89 down-regulated genes) were used to build networks of physical and functional associations in STRING. Networks were overlaid using Cytoscape. Graphical illustration of cellular localization and interactions between up-regulated genes was performed using Ingenuity Pathway Analysis (QIAGEN).

### Cell lines and antibodies

UMSCC1, UMSCC2, UMSCC43 and UMSCC47 cells were kindly provided by Dr. Thomas Carey (University of Michigan, Ann Arbor, MI). Cells were grown in Dulbecco’s Modified Eagle Medium (DMEM; Thermo Fisher Scientific) supplemented with 10% (v/v) fetal bovine serum (FBS; Thermo Fisher Scientific), non-essential amino acids (100 nmol/L; Thermo Fisher Scientific), penicillin and streptomycin (100 mg/mL; Thermo Fisher Scientific) and maintained at 37° C with 5% CO_2_.

### Spheroid invasion assay

UMSCC1 cells were cultured and transferred to Corning spheroid microplates (5,000 cells per well in 200 µl of growth media) and maintained at 37° C with 5% CO_2_ for 24 hours. Following the incubation period, growth media was removed (160 µl) and replaced with Geltrex (100 µl; 15.7 mg/ml; Life Technologies) containing 3% DQ-Green BSA (Thermo Fisher). Growth media containing TNFα (10 ng/ml) or GM6001 (25 µM) were added and spheroids were maintained at 37° C with 5% CO_2_ for 17 days. Images of each spheroid were taken by the same operator using the same spinning disk confocal microscope (Quorum spinning disk confocal, Leica DMIRE2) using z-stacks (2 µm slices) every 24 hours for 17 days. We have used the exact same imaging settings for all replicates. To determine the degradation volume, we quantified all voxels with a positive green fluorescence signal in the field of view and reported the sum of the volume of these pixels as the total degradation volume. Image analysis was performed using Volocity 6.3, and the spheroid tumor volume and Geltrex degradation volume was calculated.

### Matrix degradation and invadopodia analysis

Invadopodia assays and matrix degradation were performed as described earlier [14]. Briefly, UMSCC1 cells (50,000) were plated on Alexa Fluor-488 gelatin matrix-coated Mattek dishes (10 mm) and incubated for 24 hours with or without treatments and inhibitors described. After 24 hours, cells were fixed in 3.7% Paraformaldehyde (PFA) for 20 minutes followed by immunocytochemistry for cortactin and Tks5 as described before [14].

### mRNA sequencing

UMSCC1 cells were plated in 6-well plates (500,000 per well) for 24 hours followed by cell lysis and RNA isolation. The TNFα group was stimulated with TNFα (10 ng/ml) for 24 hours. RNA was isolated using Invitrogen RNA PureLink HiPure following the manufacturer’s protocol. RNA libraries were created using TruSeq RiboZero Gold sample preparation kit. Sequencing was performed using a paired-end 75bp with 35 million reads per sample (Illumina NextSeq500). Overall read quality was checked using FASTQC v.0.11.2. The raw sequence data, in the form of FASTQ files, were aligned to the human genome (hg19, iGenome GTF definition file) using the BOWTIE/TOPHAT pipeline (BOWTIE v2.2.6, TOPHAT 2.1.0). Transcript assembly, abundance estimation, and tests for differential regulation and expression were done using CUFFLINKS (v2.2.1).

### Patient survival analysis

RNA sequencing data for 519 head and neck squamous cell carcinoma (HNSCC) samples were obtained from The Cancer Genome Atlas (TCGA) [15] and analyzed with cBioPortal tools [16] and MATLAB (The Mathworks, Inc., MA, USA). The analyzed dataset contains mRNA expression Z-scores (RNA-Seq V2 RSEM) computed as the relative expression of an individual gene and tumor to the expression distribution of all samples that are diploid for the gene. HNSCC samples are annotated with Disease Free Survival (DFS) and Overall Survival (OS), and censorship status. HPV-positive patients were excluded. Samples were split into high and low expressing groups based upon mRNA expression Z-scores. The Z-score cut-off value for a given gene was defined as that which gave the lowest *p*-value. Associations between Z-scores and patient survival (DFS and OS) were assessed by Kaplan–Meier time-event curves and Mantel-Haenszel hazard ratios using an implementation of Kaplan–Meier log-rank testing from MATLAB Exchange.

### Invadopodia and matrix degradation

The cells were plated for 24 hours in an Alexa 488 or 405 stained gelatin matrix in 10% DMEM. The samples were then blocked in 1% bovine serum albumin (BSA) and 1% FBS for 1 hour and incubated with primary antibodies cortactin (1:300) and Tks5 (1:50) or phospho-cortactin (pY421) (1:100) or TNFR1 (1:100) in 1% BSA, 1%FBS for 1 hour. Cells were then washed with phosphate-buffered saline (PBS) three times followed by secondary antibodies anti-mouse Alexa Fluor-555 (1:300) and anti-rabbit Alexa Fluor-647 (1:300) for 1 hour at room temperature. Matrix degradation and invadopodia formation were analyzed using spinning disk confocal microscopy (Quorum spinning disk confocal, Leica DMIRE2). Image analysis was performed using Volocity 6.3, and the number of invadopodia was calculated as the number of colocalizing cortactin and Tks5 spots divided by the cell area. For ratio imaging calculation, we have analyzed cells stained for both total cortactin (red channel) and P421Y Cortactin (Far red). Invadopodia were detected using a semi-automated protocol optimized to detect cortactin spots in the cytoplasm. We then calculated the mean fluorescence intensity (MFI) of P421Y cortactin at the invadopodia divided by the MFI of total cortactin in the same pixel. The degradation area was calculated as the sum of the loss-of-fluorescence areas in the field as described previously [14]. Where indicated, UMSCC1 cells were transfected with Dharmacon Smartpools TNFR1 SiRNA using Neon electroporation (Thermo Fisher). After 72 h incubation the cells were used in Transwell assays and invadopodia assays. We achieved a ∼70% KD of TNFR1 in the 3 experiments performed.

### Western blot analysis

UMSCC1 cells were plated on 6-cm dishes and incubated at 37° C for 24 hours with or without treatments and inhibitors. Cells were treated with TNFα (10 ng/ml) and/or IL-8 (10 ng/ml). Inhibitors used individually or in combination were: Src Kinase Inhibitor I (0.1 µM), LY-294,002 hydrochloride (20 µM), Erlotinib Hydrochloride (5 µM), NFκB Inhibitor (30 µg/ml), and Anti-TNFα (5 mg/mL) antibodies. Specimens were lysed at 4° C with Laemmli sample buffer, sonicated for 5 seconds, boiled for 10 minutes, and subjected to 8% SDS–PAGE. Membranes were then blocked with Odyssey Blocking Buffer (TBS) (LI-COR Biosciences) for 1 hour followed by immunoblotting with rabbit AKT1 (phospho S473) antibody (1:2000) and mouse AKT (1:300) in Odyssey Blocking Buffer (LI-COR Biosciences) at 4° C overnight. Membranes were then washed three times for 10 minutes with TBS-T and incubated for 1 hour at room temperature with LI-COR secondary antibodies anti-mouse-IRDye 680RD and anti-rabbit-IRDye 800CW in 5% BSA Tris-buffered saline-Tween (TBS-T). Western blot membranes were read using the LICOR Odyssey infrared imaging system. LI-COR Image Studio 3.1.4 was used for densitometry analysis. Results are presented as the ratio between phosphorylated AKT and total AKT in the same blot.

### Transwell invasion assay

Transwell assays were performed as described previously [10]. In summary, UMSCC1 cells (50,000) were resuspended in 200 µl 0.5% FBS/DMEM with or without treatments and inhibitors and plated in the upper chamber of Matrigel-coated transwell membranes inserts (8.0-µm; BD invasion). Cells were treated with TNFα (10 ng/ml) and/ or IL-8 (10 ng/ml). Inhibitors used individually or in combination were: Src Kinase Inhibitor I (0.1 µM), LY-294,002 hydrochloride (20 µM), Erlotinib Hydrochloride (5 µM), NFκB Inhibitor (30 µg/ml), and Anti-TNFα (5 mg/mL) antibodies. The bottom chamber was filled with 1 ml 10% FBS/ DMEM. Cells were and allowed to invade for 24 h followed by fixation in 3.7% PFA and stained with DAPI for 30 min. Intact membranes were imaged using Cytation 3 cell imager (Biotek). Cell invasion was calculated as the number of cells at the bottom surface of the membrane after the analysis of 15 low power fields per sample.

## ELISA

The ELISA experiments followed the manufacturer’s protocol. ELISA kits for MMP9 and TNFα were purchased from Life technologies (Novex). UMSCC1, UMSCC2, UMSCC43 or UMSCC47 cells (20,000) were cultured in 2 mL of DMEM culture media for 24 hours. Supernatants were collected and centrifuged at 400 rcf for 5 minutes to remove cells and stored at –80° C.

### Statistical analysis

One-way analysis of variance (ANOVA) with a Dunnett’s post-hoc test or two-way ANOVA followed by Bonferroni post-hoc test was performed using GraphPad Prism 7.00 (GraphPad Software, Inc.). Differences were considered statistically significant if *P* > 0.05 has defined as ^*^P < 0.05; ^**^P < 0.01; and ^***^P < 0.001. Error bars represent standard error of the mean (SEM).

## SUPPLEMENTARY MATERIALS FIGURES AND TABLES






