# Supplementary material for: Oral inflammation promotes oral squamous cell carcinoma invasion
Source: Oncotarget. 2018 Jun 26;9(49):29047–63. doi: 10.18632/oncotarget.25540 (PMC6044370; doi:10.18632/oncotarget.25540)
Supplement: Supplementary file 1 [file oncotarget-09-29047-s001.pdf]

Oral inflammation promotes oral squamous cell carcinoma invasion

SUPPLEMENTARY MATERIALS

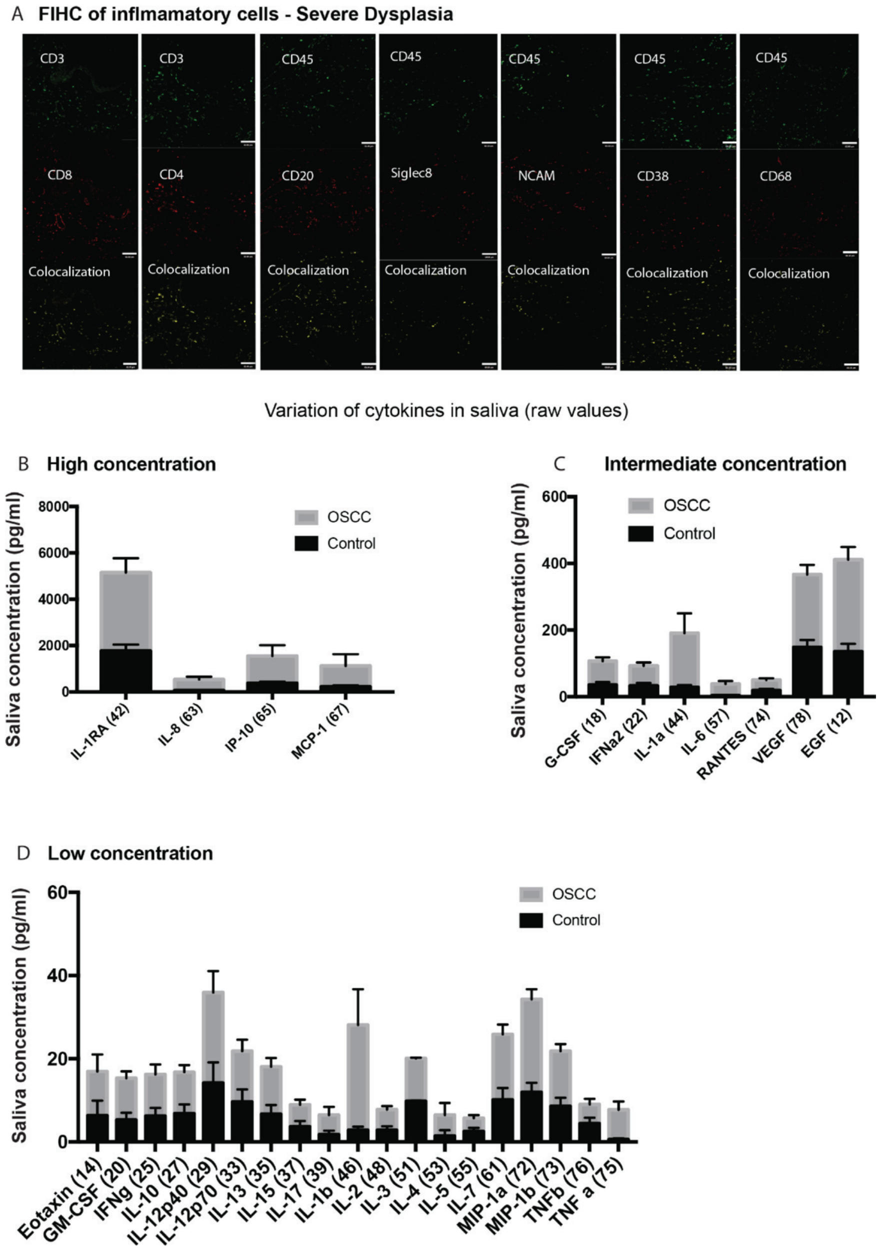

Supplementary Figure 1: (**A**) Representative images of patient samples with severe dysplasia demonstrating co-localization (yellow, overlay) of various antibody markers for inflammatory infiltrate (green and red). Scale bar, 60 µm. Concentration of all salivary inflammatory markers tested in control and OSCC patients divided in high concentration (over 500 pg/ml) (**B**), intermediate concentration (50–500 pg/ml) (**C**) and low concentration (up to 50 pg/ml) (**D**).

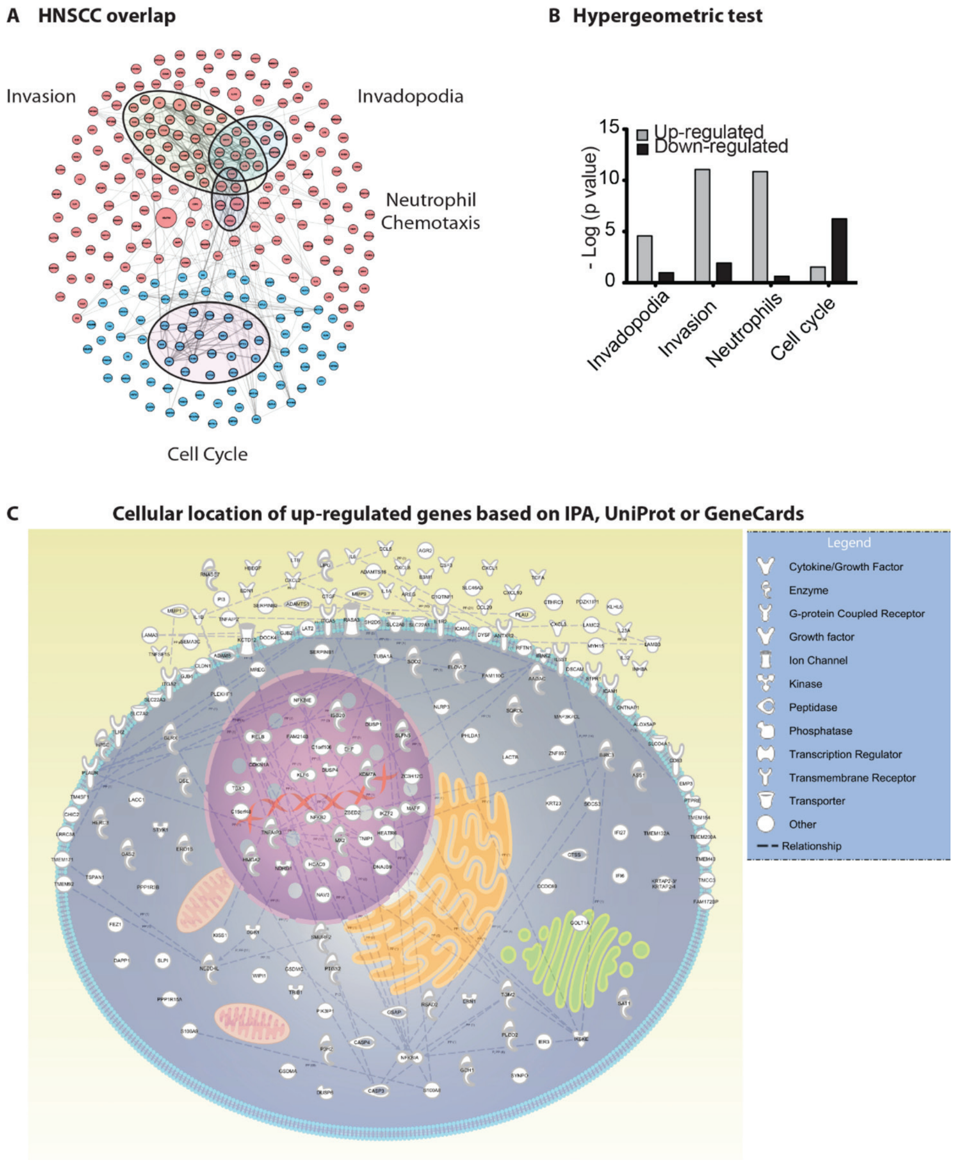

Supplementary Figure 2: (**A**) Overlap of known invasion, invadopodia and neutrophil and cell cycle genes in HNSCC with differential expressed genes identified by mRNA-seq. *p*-values were derived from a hypergeometric test in MATLAB between the number of overlapping nodes for each term within up-regulated or down-regulated genes. (**B**) Hypergeometric distribution of up-regulated and down-regulated genes from neutrophil, invasion, invadopodia, and cell cycle groups. (**C**) Graphical illustration of cellular location of up-regulated genes based on IPA, UniProt or GeneCards. Experimentally validated phosphorylation and protein-protein interactions are shown.

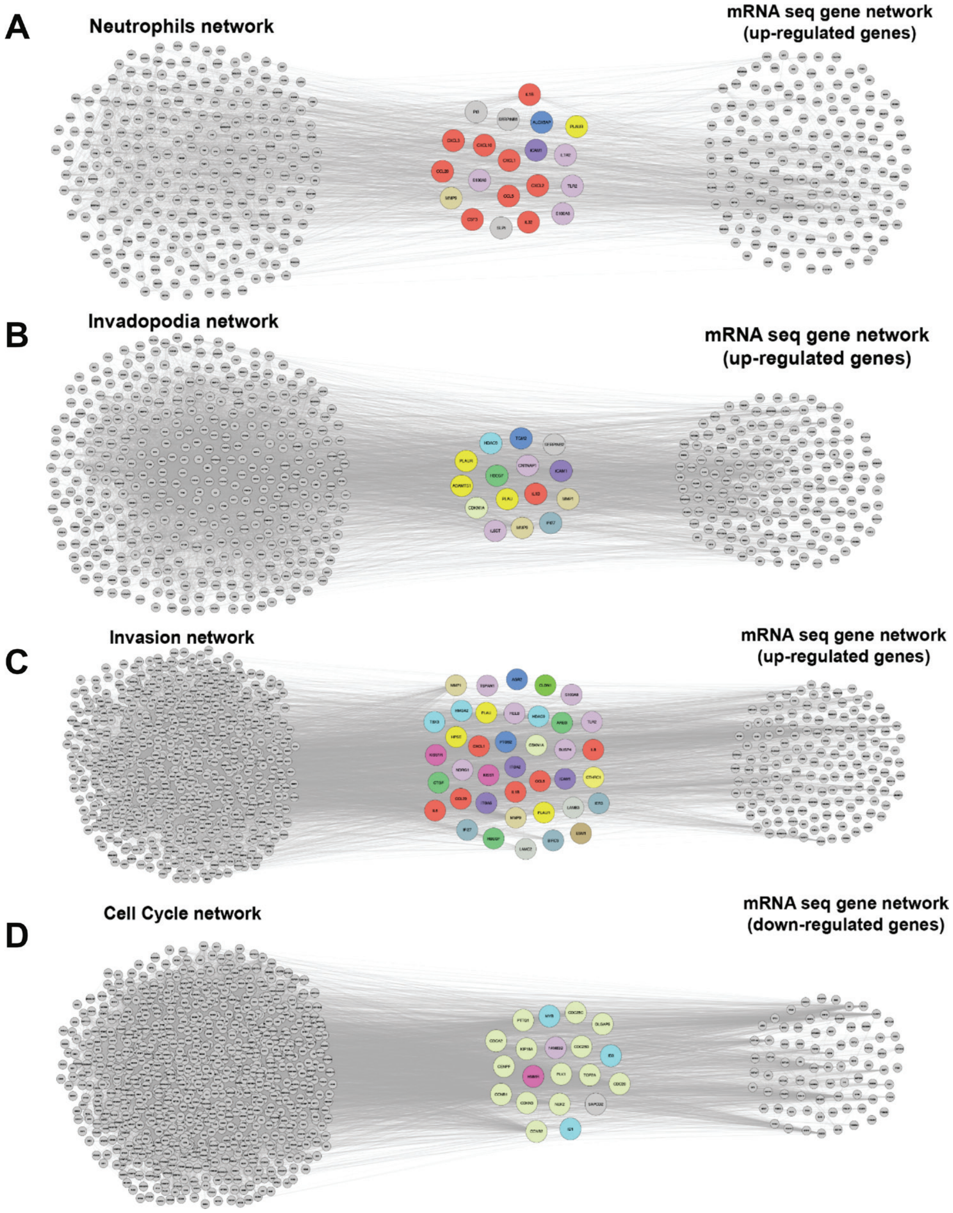

Supplementary Figure 3: Overlap of known invasion, invadopodia and neutrophil and cell cycle genes with differential expressed genes identified by mRNA-seq. Neutrophil (**A**), invadopodia (**B**), and invasion (**C**) networks with up-regulated genes and cell cycle (**D**) network with down-regulated genes were overlapped using Cytoscape. The gene lists were obtained by literature mining using GLAD4U and ALS and were used to build a physical and functional protein-protein association networks in STRING. The combined lists of common nodes were classified based on GeneCards and UniProt identified function.

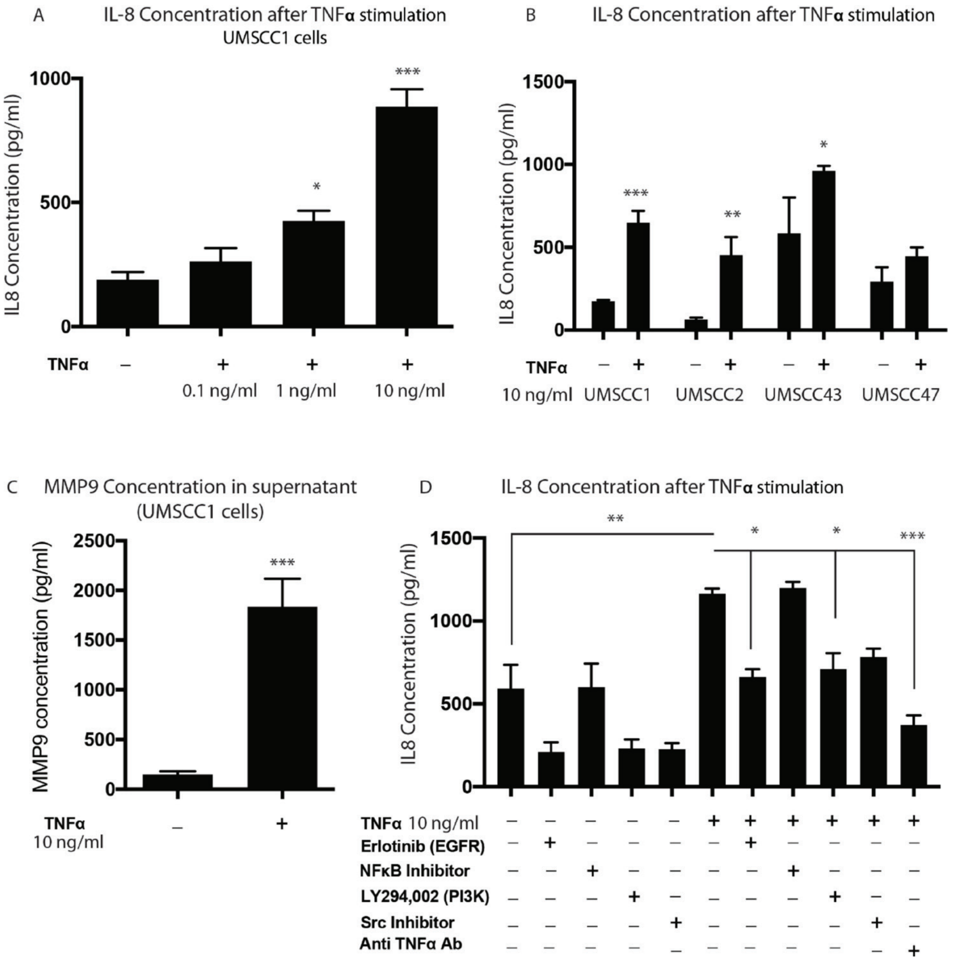

Supplementary Figure 4: (**A**) TNFα 10 ng/mL treatment of UMSCC1 leads to maximum IL8 expression. The concertation of IL8 in supernatant was measured by ELISA after stimulation of different concentrations of TNFα for 24 hours and are represented by columns ± SEM. (**B**) UMSCC1, UMSCC2, UMSCC43 and UMSCC47 cells were plated and incubated in the presence of TNFα (10 ng/mL). (**C**) The concentration of MMP9 in supernatant was measured by ELISA and are represented by columns ± SEM. (**D**) TNFα induced IL8 expression in the presence of cell signaling inhibitors. Results are based on six independent experiments. One-way ANOVA followed by Dunnett's multiple comparison test: $^{*}P < 0.05$; $^{**}P < 0.01$; $^{***}P < 0.001$.

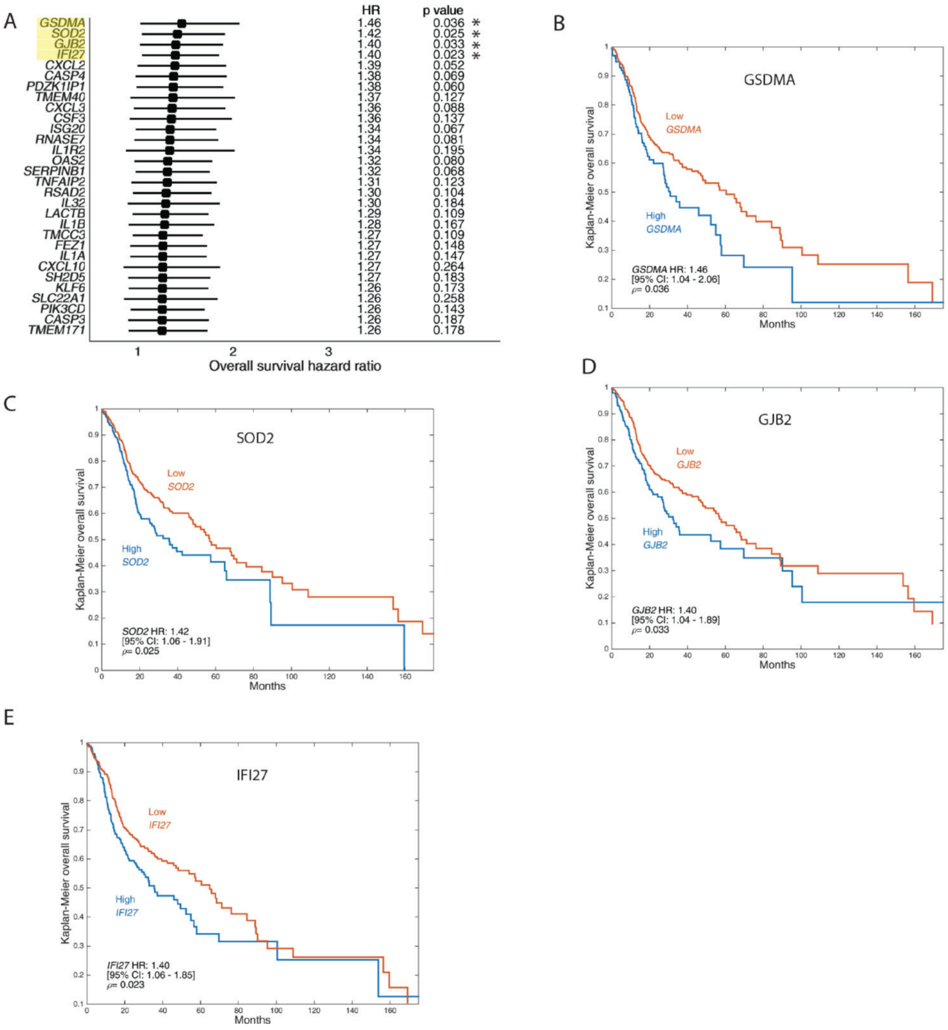

Supplementary Figure 5: Up-regulation of invasion related genes correlated with poor OSCC patient overall survival. (**A**) Forest plots of top 30 overall survival genes obtained by comparing the up-regulated genes that appeared by mRNA sequencing data to TCGA database as above. Kaplan-Meier representation of overall survival in 519 HNSCC samples from TCGA database correlated to expression of genes gasdermin A (**B**), superoxide dismutase 2 (**C**), gap junction beta-2 protein (**D**), and interferon alpha inducible protein 27 (**E**).

Supplementary Table 1: Antibodies and inhibitors

Antibody	Species	Vendor	Catalog#
CD3	mouse monoclonal	Abcam	Ab699
CD4	rabbit monoclonal	Abcam	Ab133616
CD8	rabbit polyclonal	Abcam	Ab4055
CD45	mouse monoclonal	Abcam	Ab8216
CD20	rabbit monoclonal	Abcam	Ab78237
CD66b	rabbit monoclonal	Abcam	Ab197678
CD68	mouse monoclonal	DAKO	M0876
NCAM	rabbit monoclonal	Abcam	Ab75813
Siglec-8	rabbit polyclonal	Abcam	Ab38578
CD38	rabbit monoclonal	Abcam	Ab108403
Anti-E-Cadherin	rat monoclonal	Millipore	Mabt26
Cortactin	rabbit monoclonal	Abcam	Ab3333
Anti-cortactin, phospho-specific (Tyr421)	rabbit polyclonal	Millipore	AB3852
Tks5	rabbit polyclonal	Santa Cruz Biotechnology	M-300
TNFR1	rabbit Polyclonal	Abcam	ab19139
Donkey Anti-Mouse IgG H&L (Alexa fluoro-555)	donkey polyclonal	Abcam	ab150110
Donkey F(ab')2 Anti-Mouse IgG H&L (Alexa fluoro 647)	donkey polyclonal	Abcam	ab150067
Donkey F(ab')2 Anti-Rabbit IgG H&L (Alexa fluoro-568)	donkey polyclonal	Abcam	ab175694
IRDye 680RD anti-Mouse	goat	LI-COR Biosciences	925-68070
IRDye 800CW anti-Rabbit IgG (925-32211)	goat	LI-COR Biosciences	925-32211
AKT1 (phosphor S473)	rabbit monoclonal	Abcam	ab81283
AKT	mouse monoclonal	Abcam	ab54752
Anti-IL8	mouse Monoclonal	Abcam	ab18672
Anti-TNFα	mouse Monoclonal	Abcam	ab8348
**Inhibitor**			
AKT Inhibitor	-	Santa Cruz Biotechnology	sc-394003
Erlotinib Hydrochloride	-	Santa Cruz Biotechnology	sc-202154
NFκB Inhibitor	-	Santa Cruz Biotechnology	sc-3060
Src Kinase Inhibitor I	-	Santa Cruz Biotechnology	sc-204303
LY-294,002 hydrochloride	-	Santa Cruz Biotechnology	sc-215273
GM6001	-	Sigma	M5939
MMP9 inhibitor I	-	Millipore	1177749-58-4

List of antibodies and inhibitors utilized in this study.

Supplementary Table 2: Antibody combinations for inflammatory cell population identification

**Fluorescent immunohistochemistry**	CD3 + CD8 = TCD8 cells;
FIHC	CD3 + CD4 = TCD4 cells;
	CD45 + CD20 = B cells;
	CD45 + CD66b = neutrophils;
	CD45 + CD68 = macrophages;
	CD45 + Siglec8 = eosinophils;
	CD45 + NCAM = NK cells

The combination of markers used to determine the inflammatory cell populations in patient samples utilizing fluorescent immunohistochemistry (FIHC) analysis.

Supplementary Table 3: Demographic data of patients used for saliva biomarker analysis

	Research ID	Age	Gender
**OSCC**	1	90	M
	2	83	M
	3	62	F
	4	74	F
	5	71	F
	6	82	F
	7	43	F
	8	68	F
	9	73	F
	10	60	M
	11	75	M
	12	35	F
	13	82	F
	14	48	F
	15	81	M
	16	53	M
	17	64	M
**Control**	1	28	M
	2	34	M
	3	60	F
	4	63	F
	5	54	F
	6	40	M
	7	57	F
	8	55	F
	9	57	F
	10	61	F
	11	53	F
	12	60	M
	13	34	F

The sex, age, date of saliva sample acquisition of 30 patients (17 cancer patients and 13 control patients without cancer or significant oral diseases) identified by their research ID.

Supplementary Table 4: Gene classification of up-regulated genes following TNFα stimulation. TNFα groups was stimulated with TNFα (10 ng/ml) for 24 hours. The data represents 3 independent observations per group with minimum two-fold gene expression change. $P < 0.05$. See Supplementary_Table_4

Supplementary Table 5: Gene classification of down-regulated genes following TNFα stimulation. TNFα groups was stimulated with TNFα (10 ng/ml) for 24 hours. The data represents 3 independent observations per group with significant decreased gene expression change. $P < 0.05$. See Supplementary_Table_5
