# Supplementary material for: Oral inflammation promotes oral squamous cell carcinoma invasion
Source: Oncotarget. 2018 Jun 26;9(49):29047–63. doi: 10.18632/oncotarget.25540 (PMC6044370; doi:10.18632/oncotarget.25540)
Supplement: Supplementary file 2 [file oncotarget-09-29047-s002.pdf]

Gene	Description	Classification	p_value	Fold change
HEATR6	HEAT Repeat Containing 6 (Amplified in Breast Cancer protein 1)	Unknown	5.00E-05	38.27797584
CXCL10	C-X-C Motif Chemokine Ligand 10	Cytokines and chemokines	5.00E-05	15.94104036
CSF3	Colony Stimulating Factor 3	Cytokines and chemokines	5.00E-05	15.01744345
IL1R2	Interleukin 1 Receptor Type 2	Signal transduction	5.00E-05	14.57615539
MMP9	Matrix Metallopeptidase 9	Matrix metalloproteinase	5.00E-05	12.57498775
IL8	C-X-C Motif Chemokine Ligand 8	Cytokines and chemokines	5.00E-05	12.55720946
ESM1	Endothelial Cell Specific Molecule 1	Angiogenesis	5.00E-05	11.79933002
C15orf48	Chromosome 15 Open Reading Frame 48	Unknown	5.00E-05	11.06708855
IL6	Interleukin 6	Cytokines and chemokines	5.00E-05	10.42478069
CXCL2	C-X-C Motif Chemokine Ligand 2	Cytokines and chemokines	5.00E-05	8.973566295
CCL20	C-C Motif Chemokine Ligand 20	Cytokines and chemokines	5.00E-05	8.239765975
IL32	Interleukin 32	Cytokines and chemokines	5.00E-05	7.254775008
SOD2	Superoxide Dismutase 2, Mitochondrial	Metabolism	5.00E-05	7.164987224
ICAM4	Intercellular Adhesion Molecule 4	Cell adhesion	5.00E-05	7.088417636
CXCL1	C-X-C Motif Chemokine Ligand 1	Cytokines and chemokines	5.00E-05	6.620956504
TNFSF15	Tumor Necrosis Factor Superfamily Member 15	Programmed cell death	5.00E-05	6.501874315
KRTAP2-3	Keratin Associated Protein 2-3	Cytoskeletal protein	5.00E-05	6.159735703
TNFAIP3	TNF Alpha Induced Protein 3	Protein ubiquitination	5.00E-05	6.123600166
SERPINB2	Serpin Family B Member 2	Protease inhibitor	5.00E-05	6.037214165
HBEGF	Heparin Binding EGF Like Growth Factor	Growth factor	5.00E-05	6.032909499
CDKN1A	Cyclin Dependent Kinase Inhibitor 1A	Cell division cycle	5.00E-05	5.565557824
INHBA	Inhibin Beta A Subunit	Growth factor	5.00E-05	5.453328957
PI3	Peptidase Inhibitor 3	Protease inhibitor	5.00E-05	5.386114914
ALOX5AP	Arachidonate 5-Lipoxygenase Activating Protein	Metabolism	5.00E-05	5.37421549
MMP1	Matrix Metallopeptidase 1	Matrix metalloproteinase	5.00E-05	5.331592242
IL23A	Interleukin 23 Subunit Alpha	Cytokines and chemokines	5.00E-05	5.262495573
BIRC3	Baculoviral IAP Repeat Containing 3	Programmed cell death (inhibition)	5.00E-05	5.017830685
CXCL3	C-X-C Motif Chemokine Ligand 3	Cytokines and chemokines	5.00E-05	4.96408716
CLDN1	Claudin 1	Cell-cell adhesion	5.00E-05	4.699377634
ICAM1	Intercellular Adhesion Molecule 1	Cell adhesion	5.00E-05	4.662309967
GSDMA	Gasdermin A	Vesicular transport	5.00E-05	4.603680982
EDN1	Endothelin 1	Angiogenesis	5.00E-05	4.368072491
S100A9	S100 Calcium Binding Protein A9	Signal transduction	5.00E-05	4.334464789
ADAM8	ADAM Metallopeptidase Domain 8	Matrix metalloproteinase	5.00E-05	4.261826978
ZBED2	Zinc Finger BED-Type Containing 2	Transcription	5.00E-05	4.227316582
DOCK4	Dedicator Of Cytokinesis 4	Cell-cell adhesion	0.00085	4.225082095
AADAC	Arylacetamide Deacetylase	Metabolism	5.00E-05	4.127267896
LTB	Lymphotoxin Beta	Cytokines and chemokines	5.00E-05	4.04362989
IL1B	Interleukin 1 Beta	Cytokines and chemokines	5.00E-05	3.990107205
C1QTNF1	C1q And Tumor Necrosis Factor Related Protein 1	Unknown	5.00E-05	3.803656751
PDZK1IP1	PDZK1 Interacting Protein 1	Unknown	5.00E-05	3.768685998
S100A8	S100 Calcium Binding Protein A8	Signal transduction	5.00E-05	3.726087603
PLOD2	Procollagen-Lysine,2-Oxoglutarate 5-Dioxygenase 2	ECM formation	5.00E-05	3.715939887
DSCAM	DS Cell Adhesion Molecule	Cell adhesion (neuronal)	5.00E-05	3.656421366
SLPI	Secretory Leukocyte Peptidase Inhibitor	Protease inhibitor	5.00E-05	3.562088803
SLC22A3	Solute Carrier Family 22 Member 3	Ion transporter (neuronal)	0.00095	3.512341006
LAMA3	Laminin Subunit Alpha 3	ECM formation	5.00E-05	3.411314623
IL1A	Interleukin 1 Alpha	Cytokines and chemokines	5.00E-05	3.405882942
LAMB3	Laminin Subunit Beta 3	ECM formation	5.00E-05	3.329645905
KCTD12	Potassium Channel Tetramerization Domain Containing 12	Ion transporter (neuronal)	5.00E-05	3.304352023
PTGS2	Prostaglandin-Endoperoxide Synthase 2	Metabolism	5.00E-05	3.292581454
PLAU	Plasminogen Activator, Urokinase	ECM degradation	5.00E-05	3.282443151
SLC2A6	Solute Carrier Family 2 Member 6	Glucose transporter	5.00E-05	3.26822183
DAPP1	Dual Adaptor Of Phosphotyrosine And 3-Phosphoinositides 1	Signal transduction	5.00E-05	3.21387388
NAV3	Neuron Navigator 3	Signal transduction (neuronal)	5.00E-05	3.20430376

SERPINB1	Serpin Family B Member 1	Protease inhibitor	5.00E-05	3.203094802
IRAK2	Interleukin 1 Receptor Associated Kinase 2	Signal transduction	5.00E-05	3.198203149
TMEM171	Transmembrane Protein 171	Unknown	5.00E-05	3.169364776
TM4SF1	Transmembrane 4 L Six Family Member 1	Signal transduction	5.00E-05	3.138539567
GJB2	Gap Junction Protein Beta 2	Cell-cell adhesion	5.00E-05	3.134291363
MX2	MX Dynamin Like GTPase 2	Signal transduction	5.00E-05	3.133894651
LAMC2	Laminin Subunit Gamma 2	ECM formation	5.00E-05	3.074565633
GSDMC	Gasdermin C	Programmed cell death	5.00E-05	3.033137267
PLAUR	Plasminogen Activator, Urokinase Receptor	ECM degradation	5.00E-05	3.003023991
TLR2	Toll Like Receptor 2	Signal transduction	5.00E-05	2.976070404
DUSP1	Dual Specificity Phosphatase 1	Signal transduction	5.00E-05	2.920936129
FAM214B	Family With Sequence Similarity 214 Member B	Unknown	5.00E-05	2.918916912
KRT23	Keratin 23	Cytoskeletal protein	0.00015	2.874876711
SLC22A1	Solute Carrier Family 22 Member 1	Ion transporter	5.00E-05	2.846227161
KISS1	KiSS-1 Metastasis-Suppressor	Cytoskeletal signaling	5.00E-05	2.839435436
ADAMTS1	ADAM Metallopeptidase With Thrombospondin Type 1 Motif 1	ECM degradation	5.00E-05	2.827328912
SOCS3	Suppressor Of Cytokine Signaling 3	Signal transduction	5.00E-05	2.812031289
TNIP1	TNFAIP3 Interacting Protein 1	Signal transduction	5.00E-05	2.790494775
ASS1	Argininosuccinate Synthase 1	Metabolism	0.0001	2.780131743
GLRX	Glutaredoxin	Metabolism	5.00E-05	2.773407635
TRIB1	Tribbles Pseudokinase 1	Signal transduction	5.00E-05	2.766407663
PIK3IP1	Phosphoinositide-3-Kinase Interacting Protein 1	Signal transduction	5.00E-05	2.756010345
ITGA5	Integrin Subunit Alpha 5	Cell adhesion	5.00E-05	2.702731069
TMEM154	Transmembrane Protein 154	Unknown	5.00E-05	2.695093977
TBX3	T-Box 3	Transcription	5.00E-05	2.674944798
C21orf7	Chromosome 21 Open Reading Frame 7	Unknown	5.00E-05	2.669508893
DNAJB9	DnaJ Heat Shock Protein Family (Hsp40) Member B9	Programmed cell death (inhibition)	5.00E-05	2.667574959
TMEM200A	Transmembrane Protein 200A	Unknown	5.00E-05	2.645095649
CCL5	C-C Motif Chemokine Ligand 5	Cytokines and chemokines	5.00E-05	2.626961723
FAM172BP	Family With Sequence Similarity 172 Member B, Pseudogene	Unknown	0.00025	2.613718223
AREG	Amphiregulin	Growth factor	5.00E-05	2.608005659
NFKBIE	NFKB Inhibitor Epsilon	Signal transduction	5.00E-05	2.600687806
SLFN5	Schlafen Family Member 5	Signal transduction	5.00E-05	2.59251939
CD83	CD83 Molecule	Signal transduction	5.00E-05	2.587798557
ISG20	Interferon Stimulated Exonuclease Gene 20	Anti viral response	0.0007	2.580020113
PLEKHF1	Pleckstrin Homology And FYVE Domain Containing 1	Programmed cell death	0.0009	2.578239345
RNASE7	Ribonuclease A Family Member 7	Anti microbial response	5.00E-05	2.576139485
ERN1	Endoplasmic Reticulum To Nucleus Signaling 1	Programmed cell death	5.00E-05	2.555945856
SLC7A2	Solute Carrier Family 7 Member 2	Ion transporter	5.00E-05	2.537625042
RELB	RELB Proto-Oncogene, NF-KB Subunit	Signal transduction	5.00E-05	2.535570074
HDAC9	Histone Deacetylase 9	Transcription (epigenetic)	5.00E-05	2.535425587
WIPI1	WD Repeat Domain, Phosphoinositide Interacting 1	Signal transduction	5.00E-05	2.529514745
SMURF2	SMAD Specific E3 Ubiquitin Protein Ligase 2	Protein ubiquitination	5.00E-05	2.523471988
LACC1	Laccase Domain Containing 1	Unknown	5.00E-05	2.519741646
GOLT1A	Golgi Transport 1A	Vesicular transport	5.00E-05	2.505115216
SQRDL	Sulfide Quinone Reductase-Like	Metabolism	5.00E-05	2.49473762
DUSP4	Dual Specificity Phosphatase 4	Signal transduction	5.00E-05	2.492702254
TMEM92	Transmembrane Protein 92	Unknown	5.00E-05	2.483101536
CASP3	Caspase 3	Programmed cell death	5.00E-05	2.431630304
SAT1	Spermidine/Spermine N1-Acetyltransferase 1	Metabolism	5.00E-05	2.429091068
KLF6	Kruppel Like Factor 6	Transcription	5.00E-05	2.411014244
TMEM132A	Transmembrane Protein 132A	Embryonic development (brain)	0.001	2.404409955
ITGA2	Integrin Subunit Alpha 2	Cell adhesion	5.00E-05	2.398082434
NDRG1	N-Myc Downstream Regulated 1	Signal transduction	5.00E-05	2.389064071
PION	Gamma-Secretase Activating Protein (GSAP)	Signal transduction (neuronal)	0.00015	2.388536013
SLCO4A1	Solute Carrier Organic Anion Transporter Family Member 4A1	Ion transporter	0.0002	2.356665112

IFI6	Interferon Alpha Inducible Protein 6	Programmed cell death	5.00E-05	2.348296907
RSAD2	Radical S-Adenosyl Methionine Domain Containing 2	Anti viral response	5.00E-05	2.340981837
ADAMTS16	ADAM Metallopeptidase With Thrombospondin Type 1 Motif 16	Matrix metalloproteinase	0.00035	2.340574483
NLRP3	NLR Family Pyrin Domain Containing 3	Programmed cell death	5.00E-05	2.340148092
NFKB2	Nuclear Factor Kappa B Subunit 2	Signal transduction	5.00E-05	2.332272175
LEPREL1	Prolyl 3-Hydroxylase 2	ECM formation	5.00E-05	2.326985006
NFKBIA	NFKB Inhibitor Alpha	Signal transduction	5.00E-05	2.315126291
ZC3H12C	Zinc Finger CCCH-Type Containing 12C	Signal transduction	5.00E-05	2.30293276
MAFF	MAF BZIP Transcription Factor F	Transcription	5.00E-05	2.298249289
CHIC2	Cysteine Rich Hydrophobic Domain 2	Vesicular transport	5.00E-05	2.295867144
CASP4	Caspase 4	Programmed cell death	5.00E-05	2.292008778
DUSP6	Dual Specificity Phosphatase 6	Signal transduction	5.00E-05	2.285890586
LRRC38	Leucine Rich Repeat Containing 38	Ion transporter	0.0002	2.269682628
SYNPO	Synaptopodin	Actin binding	0.0002	2.266511565
ZNF697	Zinc Finger Protein 697	Transcription	5.00E-05	2.264867154
GJB4	Gap Junction Protein Beta 4	Cell-cell adhesion	0.00045	2.264853762
TGFA	Transforming Growth Factor Alpha	Growth factor	5.00E-05	2.26119935
DSE	Dermatan Sulfate Epimerase	Metabolism	5.00E-05	2.256186172
MYH15	Myosin Heavy Chain 15	Cytoskeletal signaling	0.0006	2.245735442
CCDC69	Coiled-Coil Domain Containing 69	Signal transduction	5.00E-05	2.245433201
ANTXR2	Anthrax Toxin Receptor 2	Cell adhesion	0.00025	2.233102104
PHLDA1	Pleckstrin Homology Like Domain Family A Member 1	Programmed cell death	5.00E-05	2.227716043
GCH1	GTP Cyclohydrolase 1	Metabolism	5.00E-05	2.227460399
LAT2	Linker For Activation Of T-Cells Family Member 2	Signal transduction	0.001	2.217578376
EMP3	Epithelial Membrane Protein 3	Cell-cell adhesion	0.0004	2.214503553
JHDM1D	Lysine Demethylase 7A	Transcription (epigenetic)(brain)	5.00E-05	2.212418385
SLC46A3	Solute Carrier Family 46 Member 3	Ion transporter	0.00045	2.212374248
C1orf106	Chromosome 1 Open Reading Frame 106	Unknown	5.00E-05	2.208275493
STYK1	Serine/Threonine/Tyrosine Kinase 1	Signal transduction	0.0001	2.191483742
IFI27	Interferon Alpha Inducible Protein 27	Programmed cell death	5.00E-05	2.181173826
IKBKE	Inhibitor Of Kappa Light Polypeptide Gene Enhancer In B-Cells, Kina	Signal transduction	0.00015	2.164670884
AGR2	Anterior Gradient 2, Protein Disulphide Isomerase Family Member	Metabolism	0.0007	2.159479975
SGK1	Serum/Glucocorticoid Regulated Kinase 1	Signal transduction	5.00E-05	2.146097385
TGM2	Transglutaminase 2	Metabolism	5.00E-05	2.143600862
OAS2	2'-5'-Oligoadenylate Synthetase 2	Anti viral response	0.0001	2.138390189
PTPRE	Protein Tyrosine Phosphatase Epsilon, Receptor Type	Signal transduction	0.0001	2.126110012
FEZ1	Fasciculation And Elongation Protein Zeta 1	Cytoskeletal signaling	5.00E-05	2.123551275
DYSF	Dysferlin	Vesicular transport	0.00065	2.121115487
IER3	Immediate Early Response 3	Programmed cell death (inhibition)	5.00E-05	2.119541715
LACTB	Lactamase Beta	Metabolism	0.00015	2.117128153
RASA3	RAS P21 Protein Activator 3	Signal transduction	5.00E-05	2.111665258
IL6ST	Interleukin 6 Signal Transducer	Signal transduction	5.00E-05	2.106754807
SEMA3C	Semaphorin 3C	Development (neuronal)	5.00E-05	2.098614752
TMEM40	Transmembrane Protein 40	Unknown	0.0002	2.098309774
NEDD4L	Neural Precursor Cell Expressed, Developmentally Down-Regulated	Protein ubiquitination	5.00E-05	2.097236095
TSPAN1	Tetraspanin 1	Signal transduction	5.00E-05	2.0957848
HMGA2	High Mobility Group AT-Hook 2	Transcription	5.00E-05	2.089982322
CTHRC1	Collagen Triple Helix Repeat Containing 1	Vascular remodeling	0.0001	2.087992358
EHF	ETS Homologous Factor	Transcription	5.00E-05	2.085464088
IKZF2	IKAROS Family Zinc Finger 2	Transcription	5.00E-05	2.085405442
PPP1R3B	Protein Phosphatase 1 Regulatory Subunit 3B	Metabolism	0.00015	2.081258822
PPP1R15A	Protein Phosphatase 1 Regulatory Subunit 15A	Programmed cell death	5.00E-05	2.077019492
CTSS	Cathepsin S	Metabolism	0.00015	2.076819575
TMCC3	Transmembrane And Coiled-Coil Domain Family 3	Signal transduction (neuronal)	5.00E-05	2.071038297
LIPG	Lipase G, Endothelial Type	Metabolism	5.00E-05	2.06956324
SH2D5	SH2 Domain Containing 5	Signal transduction (neuronal)	5.00E-05	2.066854313

TNFAIP2	TNF Alpha Induced Protein 2	Vesicular transport	0.00025	2.065046979
ERO1LB	Endoplasmic Reticulum Oxidoreductase 1 Beta	Metabolism	0.0004	2.064824934
MREG	Melanoregulin	Vesicular transport	5.00E-05	2.053258852
RFTN1	Raftlin, Lipid Raft Linker 1	Signal transduction	0.0001	2.040594802
CTGF	Connective Tissue Growth Factor	Growth factor	5.00E-05	2.037646933
FAM110C	Family With Sequence Similarity 110 Member C	Microtubule organization	0.0004	2.035452133
TUBA1A	Tubulin Alpha 1a	Cytoskeletal protein	0.0006	2.025847786
S1PR1	Sphingosine-1-Phosphate Receptor 1	Cytoskeletal signaling	0.00025	2.020105374
CNTNAP1	Contactin Associated Protein 1	Signal transduction	0.0009	2.018607598
HERC3	HECT And RLD Domain Containing E3 Ubiquitin Protein Ligase 3	Protein ubiquitination	0.0003	2.013768184
ELOVL7	ELOVL Fatty Acid Elongase 7	Metabolism	5.00E-05	2.013392689
KLHL5	Kelch Like Family Member 5	Actin binding	0.00035	2.00992777
HPSE	Heparanase	ECM degradation	0.0003	2.00257565

Supplemental Table S4. Gene classification of up-regulated genes following TNFα stimulation. TNFα groups was stimulated with TNFα (10 ng/ml) for 24 hours. The data represents 3 independent observations per group with minimum two-fold gene expression change. P<0.05.
