# Supplementary material for: Oral inflammation promotes oral squamous cell carcinoma invasion
Source: Oncotarget. 2018 Jun 26;9(49):29047–63. doi: 10.18632/oncotarget.25540 (PMC6044370; doi:10.18632/oncotarget.25540)
Supplement: Supplementary file 3 [file oncotarget-09-29047-s003.pdf]

gene	Description	Classification	p_value	Fold change
CELSR2	Cadherin EGF LAG Seven-Pass G-Type Receptor 2	Cell-cell adhesion	0.00045	0.509752494
CDCA2	Cell Division Cycle Associated 2	Cell division cycle	0.00015	0.509428656
PTTG1	Pituitary Tumor-Transforming 1	Cell division cycle	0.0002	0.508455004
TNFAIP8L1	TNF Alpha Induced Protein 8 Like 1	Signal transduction	0.00095	0.507897068
KIF18A	Kinesin Family Member 18A	Cell division cycle	5.00E-05	0.501915464
KITLG	KIT Ligand	Growth factor	5.00E-05	0.495791365
MYB	MYB Proto-Oncogene, Transcription Factor	Transcription	0.00085	0.49410558
TOP2A	Topoisomerase (DNA) II Alpha	Cell division cycle	5.00E-05	0.492909074
CDC25C	Cell Division Cycle 25C	Cell division cycle	0.0002	0.491069142
EXPH5	Exophilin 5	Vesicular transport	0.00015	0.490846029
CDC20	Cell Division Cycle 20	Cell division cycle	5.00E-05	0.490607109
NEK2	NIMA Related Kinase 2	Cell division cycle	5.00E-05	0.489314748
KRT16P2	Keratin 16 Pseudogene 2	Unknown	0.00035	0.484955572
FAM83D	Family With Sequence Similarity 83 Member D	Signal transduction	5.00E-05	0.481695126
ID3	Inhibitor Of DNA Binding 3, HLH Protein	Transcription	0.00065	0.481389992
IGF2	Insulin Like Growth Factor 2	Growth factor (glucose metabolism)	0.00055	0.481311601
INS	Insulin	Growth factor (glucose metabolism)	0.00055	0.481311601
PSRC1	Proline And Serine Rich Coiled-Coil 1	Cell division cycle	5.00E-05	0.480712656
ASPM	Abnormal Spindle Microtubule Assembly	Cell division cycle	5.00E-05	0.476980285
HMMR	Hyaluronan Mediated Motility Receptor	Cytoskeletal signaling	5.00E-05	0.475562679
DEPDC1	DEP Domain Containing 1	Transcription	0.00095	0.474484898
MID1	Midline 1	Protein ubiquitination	5.00E-05	0.473761792
NRG1	Neuregulin 1	Growth factor	0.0004	0.46808087
CCNB2	Cyclin B2	Cell division cycle	5.00E-05	0.46380194
CA9	Carbonic Anhydrase 9	Metabolism	0.00065	0.463378393
ZNF618	Zinc Finger Protein 618	Transcription	0.00035	0.46149359
SERPINB13	Serpin Family B Member 13	Protease inhibitor	0.0005	0.459936941
CENPF	Centromere Protein F	Cell division cycle	5.00E-05	0.45738139
CDC25B	Cell Division Cycle 25B	Cell division cycle	5.00E-05	0.455054611
CDKN3	Cyclin Dependent Kinase Inhibitor 3	Cell division cycle	5.00E-05	0.454144736
FAM64A	Family With Sequence Similarity 64 Member A	Cell division cycle	5.00E-05	0.449934463
BDKRB2	Bradykinin Receptor B2	Signal transduction	0.0001	0.445607312
GAS2L3	Growth Arrest Specific 2 Like 3	Cytoskeletal signaling	0.0002	0.443492524
KRT80	Keratin 80	Cytoskeletal protein	0.00025	0.442195895
DLGAP5	DLG Associated Protein 5	Cell division cycle	5.00E-05	0.441119696
SLC38A4	Solute Carrier Family 38 Member 4	Amino acid transporter	0.00035	0.440920009
CCNB1	Cyclin B1	Cell division cycle	5.00E-05	0.440273307
PHGDH	Phosphoglycerate Dehydrogenase	Metabolism	5.00E-05	0.437943408
OTUD1	OTU Deubiquitinase 1	Protein ubiquitination	5.00E-05	0.437121465
KRT5	Keratin 5	Cytoskeletal protein	5.00E-05	0.434040742
SAPCD2	Suppressor APC Domain Containing 2	Unknown	5.00E-05	0.429208555
PLK1	Polo Like Kinase 1	Cell division cycle	5.00E-05	0.418584906
FGFR2	Fibroblast Growth Factor Receptor 2	Signal transduction	5.00E-05	0.41668888
RAMP1	Receptor Activity Modifying Protein 1	Signal transduction	0.00025	0.4115721
WNT10A	Wnt Family Member 10A	Signal transduction	0.0002	0.404822531
RDH10	Retinol Dehydrogenase 10	Metabolism	5.00E-05	0.402584005
THBD	Thrombomodulin	Signal transduction	5.00E-05	0.399612662
GPC1	Glypican 1	Cell adhesion	0.00015	0.39940823
EPN3	Epsin 3	Vesicular transport	0.0002	0.398961504
PBX1	PBX Homeobox 1	Transcription	5.00E-05	0.397004616
FAM46B	Family With Sequence Similarity 46 Member B	Unknown	0.00055	0.390464341
MEST	Mesoderm Specific Transcript	Metabolism	5.00E-05	0.386986629
C17orf76-AS:	LRRC75A Antisense RNA 1	Unknown	5.00E-05	0.383719101
SNORD49A	Small Nucleolar RNA, C/D Box 49A	Unknown	5.00E-05	0.383719101
SNORD49B	Small Nucleolar RNA, C/D Box 49B	Unknown	5.00E-05	0.383719101

NAV2	Neuron Navigator 2	Neuronal development	5.00E-05	0.381662351
MAP2K6	Mitogen-Activated Protein Kinase Kinase 6	Signal transduction	5.00E-05	0.370504292
GRAMD2	GRAM Domain Containing 2	Unknown	5.00E-05	0.359064131
DUSP9	Dual Specificity Phosphatase 9	Signal transduction	5.00E-05	0.359044405
PEG10	Paternally Expressed 10	Programmed cell death	5.00E-05	0.358095526
NMU	Neuromedin U	Signal transduction	5.00E-05	0.357486534
MXRA5	Matrix Remodeling Associated 5	Unknown	5.00E-05	0.355789545
KLK5	Kallikrein Related Peptidase 5	ECM formation	5.00E-05	0.351732339
TNS4	Tensin 4	Cytoskeletal signaling	0.0003	0.350926893
KRT15	Keratin 15	Cytoskeletal protein	5.00E-05	0.350289721
OLFML2A	Olfactomedin Like 2A	Cell adhesion	5.00E-05	0.349750362
UGT1A1	UDP Glucuronosyltransferase Family 1 Member A1	Metabolism	5.00E-05	0.33724594
UGT1A10	UDP Glucuronosyltransferase Family 1 Member A1	Metabolism	5.00E-05	0.33724594
UGT1A3	UDP Glucuronosyltransferase Family 1 Member A3	Metabolism	5.00E-05	0.33724594
UGT1A4	UDP Glucuronosyltransferase Family 1 Member A4	Metabolism	5.00E-05	0.33724594
UGT1A5	UDP Glucuronosyltransferase Family 1 Member A5	Metabolism	5.00E-05	0.33724594
UGT1A6	UDP Glucuronosyltransferase Family 1 Member A6	Metabolism	5.00E-05	0.33724594
UGT1A7	UDP Glucuronosyltransferase Family 1 Member A7	Metabolism	5.00E-05	0.33724594
UGT1A8	UDP Glucuronosyltransferase Family 1 Member A8	Metabolism	5.00E-05	0.33724594
UGT1A9	UDP Glucuronosyltransferase Family 1 Member A9	Metabolism	5.00E-05	0.33724594
ARSI	Arylsulfatase Family Member I	Metabolism	5.00E-05	0.330188719
CYP1A1	Cytochrome P450 Family 1 Subfamily A Member 1	Metabolism	5.00E-05	0.320665124
PINLYP	Phospholipase A2 Inhibitor And LY6/PLAUR Domair	Metabolism	0.0004	0.310711142
CRABP2	Cellular Retinoic Acid Binding Protein 2	Metabolism	5.00E-05	0.30671551
METTL7A	Methyltransferase Like 7A	Metabolism	5.00E-05	0.300517864
C10orf67	Chromosome 10 Open Reading Frame 67	Unknown	0.0001	0.290971275
NGFR	Nerve Growth Factor Receptor	Signal transduction (neuronal)	5.00E-05	0.290557603
HR	Hair Growth Associated	Transcription	5.00E-05	0.282378985
ID1	Inhibitor Of DNA Binding 1, HLH Protein	Transcription	5.00E-05	0.264518578
FAT2	FAT Atypical Cadherin 2	Cell migration	5.00E-05	0.258243903
KRT13	Keratin 13	Cytoskeletal protein	5.00E-05	0.242824539
KLK6	Kallikrein Related Peptidase 6	ECM degradation	5.00E-05	0.221782568
SYT12	Synaptotagmin 12	Vesicular transport	5.00E-05	0.218959673
KRT4	Keratin 4	Cytoskeletal protein	5.00E-05	0.156592887

Supplemental Table S5. Gene classification of down-regulated genes following TNFα stimulation. TNFα groups was stimulated with TNFα (10 ng/ml) for 24 hours. The data represents 3 independent observations per group with significant decreased gene expression change. P<0.05.
